# Constructing Mixed Density Functionals for Describing Dissociative Chemisorption on Metal Surfaces: Basic Principles

**DOI:** 10.1021/acs.jpca.3c01932

**Published:** 2023-12-05

**Authors:** Théophile Tchakoua, Tim Jansen, Youri van Nies, Rebecca F. A. van den Elshout, Bart A. B. van Boxmeer, Saskia P. Poort, Michelle G. Ackermans, Gabriel Spiller Beltrão, Stefan A. Hildebrand, Steijn E. J. Beekman, Thijs van der Drift, Sam Kaart, Anthonie Šantić, Esmee E. Spuijbroek, Nick Gerrits, Mark F. Somers, Geert-Jan Kroes

**Affiliations:** † Leiden Institute of Chemistry, Gorlaeus Laboratories, Leiden University, P.O. Box 9502, 2300 RA Leiden, The Netherlands; ‡ Department of Chemical Engineering, Delft University of Technology, 2629 HZ Delft, The Netherlands; § Faculty of Applied Engineering, University of Antwerp, Groenenborgerlaan 171, 2020 Antwerpen, Belgium

## Abstract

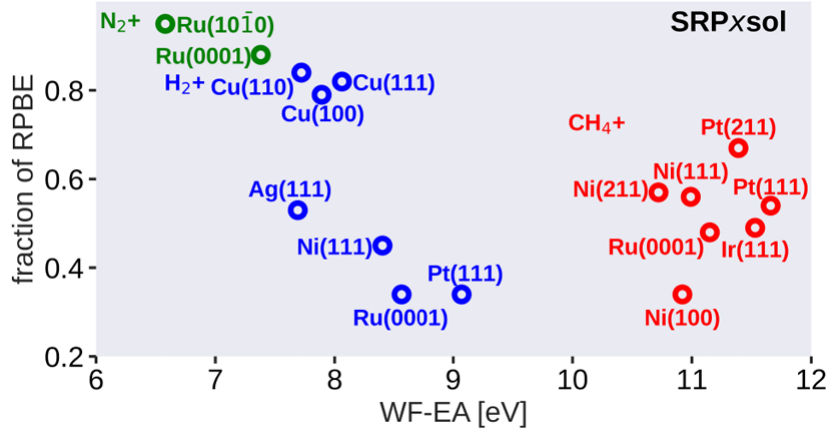

The production of a majority of chemicals involves heterogeneous catalysis at some stage, and the rates of many heterogeneously catalyzed processes are governed by transition states for dissociative chemisorption on metals. Accurate values of barrier heights for dissociative chemisorption on metals are therefore important to benchmarking electronic structure theory in general and density functionals in particular. Such accurate barriers can be obtained using the semiempirical specific reaction parameter (SRP) approach to density functional theory. However, this approach has thus far been rather ad hoc in its choice of the generic expression of the SRP functional to be used, and there is a need for better heuristic approaches to determining the mixing parameters contained in such expressions. Here we address these two issues. We investigate the ability of several mixed, parametrized density functional expressions combining exchange at the generalized gradient approximation (GGA) level with either GGA or nonlocal correlation to reproduce barrier heights for dissociative chemisorption on metal surfaces. For this, seven expressions of such mixed density functionals are tested on a database consisting of results for 16 systems taken from a recently published slightly larger database called SBH17. Three expressions are derived that exhibit high tunability and use correlation functionals that are either of the PBE GGA form or of one of two limiting nonlocal forms also describing the attractive van der Waals interaction in an approximate way. We also find that, for mixed density functionals incorporating GGA correlation, the optimum fraction of repulsive RPBE GGA exchange obtained with a specific GGA density functional is correlated with the charge-transfer parameter, which is equal to the difference in the work function of the metal surface and the electron affinity of the molecule. However, the correlation is generally not large and not large enough to obtain accurate guesses of the mixing parameter for the systems considered, suggesting that it does not give rise to a very effective search strategy.

## 1 Introduction

Transition states formed by the barriers to dissociative chemisorption (DC) can exert a high degree of rate control over the rates of heterogeneously catalyzed reactions proceeding over metal surfaces,^1,2^ such as ammonia production^3,4^ and steam reforming.^5^ It is therefore important to describe such barriers accurately. As the production of the majority of chemicals involves heterogeneous catalysis,^6^ being able to describe such barriers accurately is important. However, as discussed in several recent papers,^7,8^ it is not yet possible to use nonempirical present-day electronic structure theory to compute barriers for DC on metal surfaces with guaranteed chemical accuracy (errors ≤1 kcal/mol), although the development of an approach based on diffusion Monte Carlo certainly holds promise in this respect.^9^ Instead, success with achieving a chemically accurate description of DC on metals has so far been based on a semiempirical approach.^7,10^ Here, the specific reaction parameter (SRP) approach to density functional theory (DFT) is used to compute a potential energy surface (PES)^10−16^ or to construct forces used in direct dynamics calculations,^17−20^ and an empirical parameter in the functional used is tuned to achieve agreement between calculated and measured DC or “sticking” probabilities, as now documented extensively elsewhere.^7^ The barrier heights extracted from such work have already been collected in databases with barrier heights for DC on transition-metal surfaces.^8,21^ This endeavor is obviously important to progress with modeling heterogeneous catalysis, to testing existing and new density functionals,^8,21^ and to test first-principles methods like diffusion Monte Carlo.^9^ In this sense it is odd that databases used for benchmarking DFT,^22−26^ which with more than 30,000 papers published annually^27^ might be called the most important electronic structure method for complex systems, do not yet incorporate a database with barrier heights for DC on transition metals.

As summarized in a recent review paper^7^ and in a paper in which an extended database was published,^8^ the SRP-DFT approach has already been quite successful and has provided considerable physical insight into how DC on transition metals can be modeled with DFT. Accurate barrier heights have now been extracted for 14 systems in which H_2_, CH_4_, or N_2_ dissociate on a metal surface. For systems with a charge transfer parameter Δ*E*_CT_ = WF – EA greater than 7 eV (WF is the work function of the metal surface, and EA is the electron affinity of the molecule) the main challenge to SRP-DFT appears to find a density functional (DF) that generates the correct minimum barrier height for the system.^7^ Fortunately, and interestingly, for the systems for which the stated condition holds this can be achieved using DFs incorporating exchange from the generalized gradient approximation (GGA), even though a fraction of exact exchange is generally required for reproducing gas-phase reaction barriers.^7,8^ The success of the SRP-DFT approach for DC on metals strongly suggests,^7^ and diffusion Monte Carlo calculations on DC of H_2_ on Al(110) show,^28^ that nonempirical DFs tend to perform well at reproducing how DC barrier heights vary with geometry in a given system. The validity of minimum barrier heights and their variation with system geometry can be established through comparison of DC probabilities computed with a suitable dynamical model and method with values measured in supersonic molecular beam experiments.^7^ This statement can be underpinned^7^ with the so-called hole-model,^29^ which essentially holds that the DC probability can be computed as the fraction of geometries of the system for which the total energy exceeds the barrier height. Various strategies for developing SRP-DFs have been discussed in the recent review paper, one of which is to rely on the often-found transferability of an SRP-DF among chemically related systems.^7^

While the SRP-DFT approach has already been highly successful, it is also important to recognize that there have been some inadequacies in the approach used so far. An important shortcoming regarding the strategy of developing new SRP-DFs, which we will address here, has been that the approach to picking an expression for the SRP-DF has been rather ad hoc.^10−20^ Approaches used so far have been (i) to take a weighted average of two exchange correlation (XC) functionals within the generalized gradient approximation (GGA),^10,11^ (ii) to take a weighted average of two exchange (X) functionals within the GGA and to combine the resulting X functional with a GGA correlation (C) functional,^30^ (iii) as in (ii), but use a nonlocal C functional^14,15,19,20^ also approximately describing the attractive van der Waals interaction,^31,32^ (iv) to take a GGA exchange functional that was designed to be tunable^33^ and to combine it with nonlocal van der Waals correlation,^12,13^ and (v) to use meta-GGA functionals either with semilocal correlation^34^ or in combination with nonlocal correlation.^16^

The time is now ripe to address some basic issues in SRP functional construction, such as (i) can we use a generic expression of the density functional (DF) in such a way that the DF will usually work, and (ii) might it be possible to pick the expression in such a way that the tuning parameter (the “specific reaction parameter”) can be made to correlate with a specific property of the system. Two recent and closely related developments ensure that the time is now right. The first is that a new database of dissociative chemisorption barrier heights has recently become available, which has been called the SBH17 database.^8^ The database holds reference values of barrier heights to DC for 17 systems in which H_2_, N_2_, or CH_4_ dissociates over a metal surface. For 14 of these systems the barrier height was determined using SRP-DFT, while for 3 systems a more ad-hoc semiempirical procedure was used to extract a barrier height from a comparison between theory and experiment.^8^ In a second development^35^ it has become clear that the SRP-DFT approach based on GGA exchange functionals has its limits. So far this approach has only been successful for systems in which the charge transfer parameter Δ*E*_CT_ = WF – EA > 7 eV.^35^ Here WF is the work function of the metal, and EA is the electron affinity of the molecule. The SBH17 database therefore mostly contains systems for which this condition has been obeyed, which is the case for 16 out of the 17 systems.^8^ For systems with Δ*E*_CT_ < 7 eV the use of even one of the most repulsive GGA X DFs, i.e., RPBE,^36^ typically leads to underestimated barrier heights.^35^

Here we test several mixed DF expressions to see if we can derive generic expressions that work for all or most systems in the recently published database,^8^ to improve the toolbox of strategies aimed at deriving SRP-DFs for DC on metals. We also test the suggestion implicit in ref (35) that the fraction of the RPBE exchange needed in a mixed functional correlates with the value of the charge transfer parameter introduced above. This is also relevant to the strategy of developing new SRP-DFs^7^ and thereby extending the data available for databases: This should obviously be facilitated if a strong correlation between the fraction of RPBE exchange needed and the charge transfer parameter should exist. Our paper is set up as follows: In Section 2, the Methods Section, we give a short description of the database we use, which is essentially our previously published database with one system removed from it, in Section 2A. In Section 2A we also provide a lengthy discussion showing that high-level first-principles calculations are not yet capable of providing reference values of barrier heights for DC on metals, of how these values are instead extracted with semiempirical SRP-DFT based on experimental measurements, and what the accuracy is of the reference values in SBH17. Section 2B describes the DFs tested, and Section 2C gives computational details. Section 3 presents our results, and conclusions are drawn in Section 4.

## 2 Methods

### 2A The SBH16 Database

The DFs described in Section 2B have been tested on what we here call the SBH16 database, which is the recently described SBH17 database^8^ with the H_2_ + Pt(211) system removed from it. The reason that we left out the H_2_ + Pt(211) system described here is that the results for this system are not that different from those for the H_2_ + Pt(111) system also contained in the SBH17 system, so that not so much is to be gained by adding results for the H_2_ + Pt(211) system to the results here presented. The SBH17 database holds results for 8 H_2_ + metal surface systems (SBH16 for 7 such systems), 2 N_2_ + metal surface systems, and 7 CH_4_ + metal systems. The reference values of the barrier heights for these systems and the most important geometrical parameters determining the barrier geometry of the molecule relative to the surface are all presented in Table 2 of ref (8). Ref (8) also provides the references to the papers in which fuller descriptions of the barrier geometries and of how they were derived may be obtained.

A few details regarding the SBH17 database are important to this paper. One is that the barrier heights and geometries are in principle defined best for the 14 out of the 17 systems (13 in SBH16), for which the reference values were obtained with SRP-DFT. Results for three systems (CH_4_ + Ni(100), CH_4_ + Ru(0001), and N_2_ + Ru(101̅0)) were obtained with more ad hoc semiempirical procedures, as discussed in detail in ref (8). The result that, of the three systems for which on average the largest errors were found with the density functionals tested, two systems were among the systems for which more ad hoc semiempirical procedures were used (i.e., CH_4_ + Ru(0001) and N_2_ + Ru(101̅0)) is consistent with the lower accuracy anticipated for the ad hoc procedure (the third system for which the DFs tested were least accurate on average was H_2_ + Ag(111)). Finally, a useful number characterizing the SBH16 database is the average value of the absolute values of the barrier heights contained in it, which is 0.687 eV (15.9 kcal/mol).

For a detailed understanding of our SBH17 database, it is useful to first summarize the state of the art in computing barrier heights for dissociative chemisorption on metals with high-level methods, also comparing them to how the methods concerned perform for databases of gas-phase reactions. For the gas-phase databases we will quote results for the BH76 database (with barrier heights for 38 H atom (HTBH38) and 38 non-H atom (NHTBH38) transfer reactions)^23^ and the DBH24 database, which is a statistically relevant subset of HTB38 and 44 hydrogen-atom transfer reactions.^22^ Note that over the years modifications have been made to the reference data, and it is most appropriate to label these databases by the year in which an (updated) database appeared.^37^

The coupled-cluster with single, double, and perturbative triple particle-hole excitation operators (CCSD(T))^38^ is typically considered the gold-standard of high-level ab initio methods. This method indeed reproduces reference values of gas-phase reaction barriers with subchemical accuracy, i.e., with a mean absolute error (MAE) of 0.46 eV for the DBH24/08 database.^22^ To the best of our knowledge, this method has not yet been used in published calculations of barriers for DC on metals. However, a periodic version of CCSD(T) has been used to compute barrier heights for DC of H_2_ on Si(100), for two reaction paths.^39^ The computed activation energies (0.70 and 0.75 eV, respectively) were in good agreement with the experimental lower bound quoted for the activation energy (0.6 eV).^39^ The periodic CCSD(T) method was therefore claimed to exhibit chemical accuracy for this reaction, although it is not quite clear how this can be done on the basis of a lower bound only for the experimental activation energy.

The diffusion Monte Carlo (DMC) method^40,41^ is likewise a highly accurate first-principles method, with an MAE of 1.2 kcal/mol for the BH76 database.^7,42,43^ It has been used to compute barrier heights for the H_2_ + Cu(111),^9^ H_2_ + Mg(0001),^44^ H_2_ + Al(110),^28^ and N_2_ + Cu(111)^45^ DC reactions. Only for H_2_ + Cu(111) a comparison based on experiment and semiempirical SRP-DFT calculations has been made, and for this one system it suggested the DMC barrier height to be accurate to within almost chemical accuracy (1.6 ± 1.0 kcal/mol).^9^

Calculations on DC on metals have also been done with embedded correlated wave function (ECW) methods,^46,47^ with the correlated wave function methods used including CASPT2^48,49^ (a multireference second order perturbation theory based on a complete-active space reference function) and n-electron valence second-order perturbation theory (NEVPT2).^50^ The accuracy of these methods should be similar to that of multireference second-order Møller–Plesset perturbation theory (MRMP2), which yields an MAE of 1.4 kcal/mol for the DBH24/08 database.^51^ A potential energy surface based on embedded CASPT2 allowed DC probabilities of O_2_ dissociating on a simple metal surface (Al(111)) to be reproduced with near chemical accuracy,^52^ although the accuracy was also overestimated somewhat by only simulating the reaction of the rotational ground state.^35^ Calculations with embedded CASPT2 on DC of H_2_ on Cu(111)^53^ were less successful, producing a barrier height (0.15 eV) that fails to reproduce the semiempirical SRP-DFT value of 0.63 eV.^10^ (As explained in ref (8) in ref (53) a mistaken assessment of the quality of embedded CASPT2 was made for H_2_ + Cu(111) on the basis of an incorrect assumed value of the barrier height of only 0.05 eV.) The embedded NEVPT2 result for the barrier height (0.66 eV)^53^ is actually in much better agreement with the semiempirical SRP-DFT value (0.63 eV),^10^ but as the authors stated themselves it is not clear why the embedded CASPT2 and NEVPT2 methods should not agree for H_2_ + Cu(111).^53^ It is possible that there is a convergence problem with calculations of embedded CASPT2 and NEVPT2 on DC on transition-metal surfaces as the cost of these methods scales unfavorably with system size.

The random phase approximation (RPA),^54−56^ which may be viewed as a 5^th^ rung density functional, yields an MAE of 2.3 kcal/mol for the BH76 database,^57^ which is larger than the DMC value quoted earlier (1.2 kcal/mol). To our knowledge, the RPA has not yet been used for the calculation of barrier heights for DC on metals in published work. The RPA has been used on DC of H_2_ on Si(100), with the resulting barrier heights showing similar deviations (up to 70 meV) from periodic CCSD(T) values computed in the same work as the DMC barriers computed earlier.^39^ The RPA has been tested on a reduced version (CE10)^58^ of a database of 25 chemisorption energies on transition metals (CE25).^59^ These RPA calculations yielded an MAE of 4.8 kcal/mol for the CE10 database, but there may have been problems with the convergence of these calculations.^58^ The RPA has also been applied to the calculation of adsorption energies in specific systems,^60−63^ also with applications to systems relevant to electrochemistry.^64,65^

It is also worth mentioning a recent ONIOM-type approach.^66^ In the approach used,^67^ “high-level” and “low-level” cluster and low-level periodic calculations were used to extract chemisorption energies for a database of 25 molecule-transition metal surface systems and to extract reaction barrier heights for a subset of 5 molecule-transition metal surface systems contained in the SBH10 database. The M06 global hybrid functional^68^ was used in the high-level cluster calculations, and the dispersion-corrected PBE-D3 GGA functional^69−71^ was used in the low-level cluster and periodic calculations. The approach gave an MAE of 2.2 kcal/mol for the chemisorption energies and of only 1.1 kcal/mol for the DC barrier heights. The latter number might seem impressive. However, the approach used contains deficiencies. In ref (67) the description of the methodology suggests that they have used transition-state geometries in which the positions of the surface atoms were relaxed in the presence of the molecule, while the reference values quoted in Table 4 of this paper are all for surface geometries relaxed with respect to the vacuum. Furthermore, according to ref (67) a zero-point energy correction was applied to the activation energies, while the reference values in their Table 4, to which they compare their PBE+D3/M06 results, did not contain this correction. This means that the MAE derived is unlikely to be accurate, and we advocate that the PBE+D3/M06 approach also be tested on a larger database such as SBH17,^8^ in a correct manner. Further objections one might raise to the ONIOM-based approach taken are that clusters with different combinations of numbers of atoms and shape were used for each of the 5 face-centered cubic (fcc) DC systems (although a protocol was followed in setting up the clusters) and that the size of the clusters is subject to restrictions for magnetic metal surfaces. Furthermore, the method will be hard to validate for applications requiring potential energy surfaces, as the approach requires the molecule to be centered on the cluster in the transition state geometry corresponding to one specific impact point of the surface.

In view of the lack of accuracy of high-level first-principles and of DFs on high rungs of Jacob’s ladder of Perdew,^72^ a different approach has been taken to obtain reference values for DC on transition metals, as already alluded to above. This approach is semiempirical. All reference values in the database are ultimately based on experiment or comparisons of theory with experiment. For most cases in the SBH17 database (see ref (8)), the specific reaction parameter approach to density functional theory (SRP-DFT) was used,^7,10^ which involves, for each system considered, the development of a functional (the SRP functional or SRP-DF) that is tailored to that specific system. This is done by fitting a parameter in the SRP-DF (the specific reaction parameter or SRP) to reproduce an experimental DC curve (also called sticking curve) for that system, which is given by the DC probability (or sticking probability) as a function of the average incidence energy of the molecule^7,10^ (see below for expressions used for SRP-DFs). We use sticking probabilities extracted from supersonic molecular beam experiments rather than rate constants because the former reflect the reaction at the well-defined surface geometries on the terraces of the low-index Miller surfaces used in the experiments. Instead, reaction rates often reflect reaction at unknown surface defect geometries.^7,73^ In the approach adopted, one accepts the DF tried as an SRP-DF if the computed sticking probability curve is shifted relative to the experimental curve along the incidence energy axis by less than 1 kcal/mol, which is generally accepted as the criterion for chemical accuracy. The approach will work if the dynamical model and the dynamics method used are selected to capture the important dynamical effects in the system and if the sticking probability is computed with appropriate thermal averaging over the rovibrational states of the reacting molecule and over the distribution of incidence translational energies. Here, “model” refers to, e.g., inclusion or not of surface atom motion and/or electron–hole pair excitation, and “method” refers to the use of, e.g., quantum or quasi-classical dynamics.^7^

Because the SRP-DFT approach is not only based on experiment but generally also on a parametrized DF that is adjusted to reproduce a specific experiment, the reference values extracted for the barrier heights for DC on metals have come to be called “semi-empirical”, rather than just “experimental”. Here, semiempirical is meant in a general sense, i.e., as “involving assumptions, approximations, or generalizations designed to simplify calculation or to yield a result in accord with observation” (Merriam-Webster dictionary). Usually computed and measured sticking probabilities exhibit a similar width (or equivalently slope), and this finding^7^ and DMC and DFT calculations on DC of H_2_ on Al(110)^28^ suggest that standard DFs usually get the distribution of the barrier heights (over geometries of the system) right but not the minimum barrier height, which requires tuning of the SRP.^7^ Because the shapes of the experimental and computed sticking probabilities are usually the same, their discrepancy can be characterized by a single parameter, i.e., the energy shift of the theoretical curve relative to the measured one. This also explains why the SRP-DFT procedure used, in which only one parameter is adapted in the DF being tailored to the experiment, works so well in practice.

As already mentioned above, once the shift is less than 1 kcal/mol the parametrized DF is accepted as an SRP-DF. Particular attention is paid to whether this is true also for the lowest incidence energies, which, in view of the constant energy shift mentioned, is usually the case. The semiempirical minimum barrier height is then extracted by using an appropriate algorithm such as the nudged elastic band^74,75^ or the dimer^76^ method, either using the SRP DF directly or using an accurate global fit of the PES computed using SRP DF data and used also in the dynamics calculations. Based on the constraint we put on the energy shift of the sticking probability curves measured and computed for a specific system for the DF to be an SRP-DF, the accuracy of the reference values for the barrier heights in SBH17 that were extracted with SRP-DFT is estimated as 1 kcal/mol. Barrier heights extracted with more approximate semiempirical procedures, as was done for 3 of the 17 systems, are likely less accurate. For a detailed discussion we refer to ref (8). Minimum barrier heights have now been extracted for 14 DC on transition metals with SRP-DFT (see ref (8) for the procedures used for the other 3 systems). The accuracy target of 1 kcal/mol, as also defined some time ago as the target to be set for energies by Pople,^77^ and usually referred to as “chemical accuracy” (see, e.g., ref (78)), is a useful target to set for the performance of electronic structure methods on DC on metals: it both reflects the accuracy thought to be achieved with SRP-DFT and would seem to be coming within reach with the highest-level first-principles methods now being tested on dissociative chemisorption, as described above.

The procedure used to obtain reference values for databases of gas-phase reaction barriers^23,25,79,80^ differs from that used for databases of surface reactions in two ways. First of all, in the gas-phase case many reference values come from high-level theoretical methods, and the proportion of the data coming from theoretical methods and the level of these methods have been increasing over the years as more accurate calculations became possible.^22,37,81−83^ The method adapted may be specific to the system in the database and may involve a specific model chemistry or a so-called multilevel model chemistry.^22,77^ Here, in this context a model chemistry is usually a combination of a specific high-level ab initio method with a specific basis set.^22^ Multilevel model chemistries may employ different high-level ab initio methods and/or different basis sets and use these to extrapolate to more accurate results.^22^ To give an example, at the high end the 2008 version of the DBH24 database, DBH24/08,^22^ contains some data from so-called Weizmann-4 theory, which uses different basis sets and *ab initio* methods beyond CCSD(T) and is able to provide atomization energies with an accuracy of 0.1 kcal/mol or better.^84^

Second, databases of gas-phase reactions, and certainly the older versions of these databases, may contain values that are based on experiments or on experiments and electronic structure calculations. Again, taking the DBH24/08 database as an example, the reference data for some of the reactions were taken from a comparison of rate constants based on measurements and electronic structure calculations, using either quantum dynamics or sophisticated versions of transition state theory, like variational transition state theory.^22^ For some reasons, these reference values are usually labeled as “experimental”, though arguably there is a semiempirical flavor to the procedure used in the earlier work, as considerations from electronic structure calculations are also taken into account. We therefore conclude that, to a large extent, labeling some of the reference values in gas-phase reaction barrier databases as “experimental” rather than as “semiempirical” is a matter of semantics. We prefer to label the reference data in the SBH17 database as “semiempirical” because in the majority of cases the reference value of the minimum barrier height was derived on the basis of a semiempirical density functional adjusted to reproduce measured sticking coefficients for that reaction.

### 2B Mixed Density Functional Expressions

The XC part of DFs used as SRP-DFs has typically been taken as mixtures of the X and C components of standard XC DFs. This has the advantage that constraints enforced in constraint-based X and C DFs can also be enforced in SRP-DFs.^85^ Based on previous experience, we test the following expressions for the exchange-correlation part of the mixed DFs.
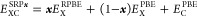
1
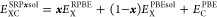
2
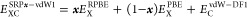
3
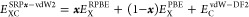
4
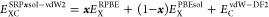
5and
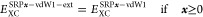
6a
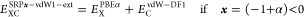
6band
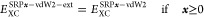
7a
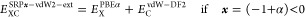
7b

The *E*_XC_^SRP**x**^ DF of eq 1 has been used to arrive at a reparameterized SRP DF for H_2_ + Cu(111),^30^ the original version being a weighted average of the RPBE^36^ and PW91^86^ DFs.^10^ In the limit ***x*** = 0 the DF defined by eq 1 corresponds to the PBE^69^ DF, and in the limit ***x*** = 1 it corresponds to the RPBE DF (which has the PBE C DF as the correlation part of its exchange-correlation functional^36^). Choosing eq 1 in attempts to derive an SRP DF for a DC-on-metal-surface system is in accordance with conventional wisdom that PBE often under-predicts and RPBE often overpredicts the barrier height for DC on a metal surface.^7^ In terms of the reduced density gradient , where *n* is the total electron density, the limiting forms of the exchange enhancement factor of the DF defined by eqs 1–7a for ***x*** = 1 (RPBE) and by eqs 1, 3, and 4 for ***x*** = 0 (PBE) may be written as follows.
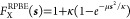
8
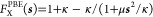
9

**Figure 1:**
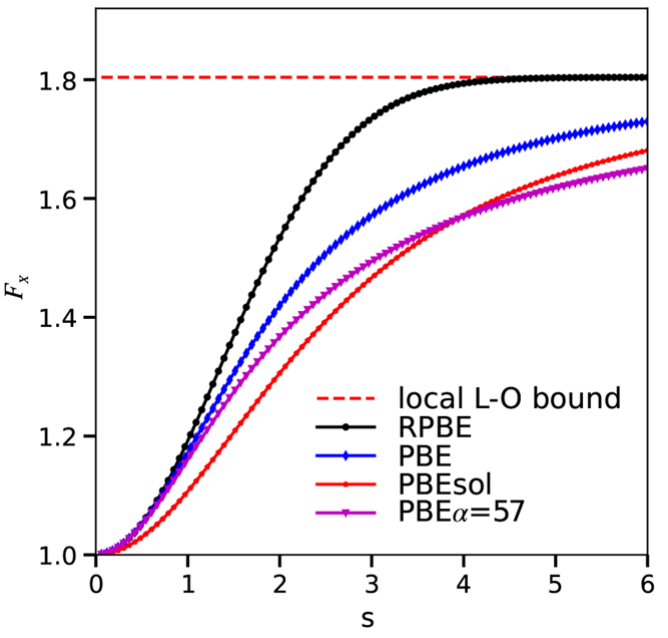
Exchange enhancement factor (*F*_*x*_) as a function of the reduced density gradient (***s***) for the RPBE^36^ (black), PBE^69^ (blue), PBEα^33^ (with α = 0.57, magenta), and PBEsol^87^ (red) functionals. The horizontal red dashed line presents the local Lieb-Oxford bound^88^ imposed in the construction of these functionals.^33,36,69,87^

Here, μ = 0.21951, and κ = 0.804.^36,69^ These exchange enhancement factors are plotted as functions of ***s*** in Figure 1.

We note that in constructing the PBE DF, the developers of this DF have built^69^ on the earlier PW91 DF.^86^ More specifically, PBE was built based on similar, though not identical, nonempirical constraints, with PBE only satisfying those constraints that the developers thought to be energetically significant.^69^ As a result of this, the exchange-correlation enhancement factors of PW91 and PBE are nearly identical for ***s*** ≤ 3.0 (see Figure 1 of ref (69)), and the exchange enhancement factors of these two DFs only start to differ for ***s*** > 4.0 (see Figure 1 of ref (89)). The region of ***s***-values for which the two DFs are in good agreement contributes most to the exchange energies of atoms^90,91^ and covalent molecular systems.^90^ Perhaps as a result of this, barriers to dissociative chemisorption on metal surfaces computed with PW91 and PBE tend to be in very good agreement with one another (differences smaller than 0.02 eV for barrier heights exceeding 0.6 eV, for instance, cf. Tables 2 (PW91 results) and 3 (PBE results) of ref (92) for CH_4_ + Ni(111), and see Figure 5 and cf. Figures 7 and 8 of ref (93) for HCl + Ag(111)). Differences between the exchange enhancement factors of PW91 and PBE that occur for large values of the reduced density gradient (***s*** > 4.0^89^) may, however, be relevant to the calculation of the exchange energy in regions where ***s*** is large and the density is low.^90,94^ In these regions exchange and correlation need to be carefully balanced to correctly describe the van der Waals interaction energy of a molecule approaching the surface,^90,94^ but this obviously also requires the use of a correlation functional that yields at least a qualitative description of the van der Waals energy.^31,32^ Furthermore, even for systems where the barrier occurs far from the surface like H_2_ + Ru(0001), differences in PW91 and PBE barrier heights tend to be small (<0.02 eV), as are differences between computed dissociative chemisorption probabilities (see Figures 4 and 9 of ref (14), respectively). Finally, we note that PW91 and PBE gave somewhat different results for monovacancy formation energies of Pt and Al, for reasons that were not well-understood, but the differences are not large.^95^

A drawback of using eq 1 is that with PBE the barrier height for DC on a metal surface may also be overestimated in specific cases, even though this DF has a negative mean signed error (MSE) of −58 meV for the SBH17 database.^8^ For this database, overestimated (though often not by much) barrier heights were observed for a few weakly activated or nonactivated H_2_-metal systems (H_2_ + Pt(111), Pt(211), and Ru(0001)), for H_2_ + Ag(111), and for a few CH_4_ + metal surface systems (CH_4_ + Ni(100), Pt(211), and Ru(0001)). To avoid this we replaced the X DF of PBE by the X DF of PBEsol,^87^ which tends to yield lower barriers, this way obtaining the *E*_XC_^SRP**x**sol^ mixed DF of eq 2. It should be noted that for ***x*** = 0 *E*_XC_^SRP**x**sol^ does not equal the PBEsol functional, which employs the same expression for the C functional as PBE but uses a different value of a coefficient in it to balance the C part of PBEsol against its X part.^87^ However, as we will show, the use of *E*_XC_^SRP**x**sol^ comes with the advantage that where necessary it yields lower barrier heights for DC on metals than *E*_XC_^SRP**x**^, and thus *E*_XC_^SRP**x**sol^ is more tunable than *E*_XC_^SRP**x**^. Below, we will call the ***x*** = 0 limit of *E*_XC_^SRP**x**sol^ PBEsolc, to distinguish it from PBEsol. The limiting ***x*** = 0 form of the exchange enhancement factor of the DFs defined by eq 2 and 5 (PBEsol) may be written as
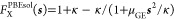
10where the form used is identical to the PBE expression (eq 9) but μ_GE_ = 0.1235 instead of μ = 0.21951 is used.^87^ This exchange enhancement factor is also plotted as a function of ***s*** in Figure 1.

A drawback of both eqs 1 and 2 is that the attractive van der Waals interaction between molecule and surface is not described with a GGA correlation functional, even though this may be necessary for weakly activated DC of H_2_ on metals (where the barrier is usually at a fairly large molecule–surface distance so that a proper description of the van der Waals interaction may be important in spite of its weakness^12,13^) or for CH_4_ dissociating on a metal surface.^19,20^ For this reason we also test the DFs of the forms *E*_XC_^SRP**x**-vdW1^ of eq 3 and *E*_XC_^SRP**x**-vdW2^ of eq 4, which contain the vdW-DF1 C functional^31^ and the vdW-DF2 C functional,^32^ respectively. The *E*_XC_^SRP**x**-vdW1^ functional has been used successfully to describe supersonic molecular beam experiments on CH_4_ + Ni(111),^19^ Pt(111),^20^ and Pt(211)^20^ and on H_2_ + Ru(0001).^14^ The *E*_XC_^SRP**x**-vdW2^ functional has been used successfully to describe H_2_ + Ru(0001)^14^ and Ni(111).^15^

The DFs described by eq 3 and 4 may have a problem similar to that of the DF described by eq 1, i.e., that the barrier height is already overestimated with ***x*** = 0, including PBE exchange only. For instance, the SRP-DF found for H_2_ + Pt(111)^12^ and Pt(211)^13^ is given by *E*_XC_^PBEα = 0.57,vdW2^ = *E*_X_^PBEα = 0.57^+*E*_C_^vdW-DF2^, where *E*_X_^PBEα = 0.57^ is the inherently tunable PBEα X DF,^33^ with α = 0.57. For the PBEα exchange DF, the exchange enhancement factor is given by
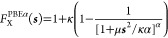
11and is plotted as a function of ***s*** in Figure 1 for α = 0.57, which is the lowest value we have used in calculations in this work. (We note that there was a typo in the equation describing *F*_X_ in the original paper describing the PBEα functional;^33^ in this equation, *x*_1_ should have appeared as *x* = μ***s***^2^.)^96^ As discussed by the developer of the PBEα X functional,^33^ PBEα = 1 corresponds to the PBE functional, while PBEα = 0.52 is very similar to the X part of the WC functional,^97^ which like PBEsol^87^ was developed with a view to a better description of the solid state. The *E*_XC_^SRP**x**-vdW2^ value with ***x*** = 0 (only PBE exchange) overestimates the barrier height for almost all systems in the SBH17 database. For this reason, we have also tested the DF *E*_XC_^SRP**x**sol-vdW2^ of eq 5, which for ***x*** = 0 consists of PBEsol exchange and the vdW-DF2 correlation functional. In this limit, this DF is expected to yield low barriers like *E*_XC_^PBEα = 0.57,vdW2^.

To increase the tunability of a mixed DF expression as given by eqs 1, 3, and 4, PBE exchange can be replaced by PBEsol exchange, as done in eq 2 to obtain a better tunable DF than the DF of eq 1, and in eq 5 to obtain a better tunable DF than the DF of eq 4. An alternative already implicitly used in the construction of SRP-DFs is to replace PBE exchange by PBEα exchange with α < 1, as done to obtain *E*_XC_^SRP**x**-vdW1-ext^ of eq 6a (which should be more tunable than *E*_XC_^SRP**x**-vdW1^ of eq 3) and to obtain *E*_XC_^SRP**x**-vdW2-ext^ of eq 7a (which should be more tunable than *E*_XC_^SRP**x**-vdW2^ of eq 4). We have not made use of the possibility of the PBEα functional to interpolate between PBE and RPBE exchange, as the PBEα X functional corresponds to the RPBE X functional only in the limit α →*∞*, which is a rather awkward limit to work with, and less preferred to a situation where switching from PBE to RPBE exchange can be performed by switching a parameter continuously from 0 to 1, as can be done in eqs 1, 3, and 4.

The DFs of eqs 1–5, 6a, and 7a have been evaluated for ***x*** = 0, *n*Δ***x*** with *n* = 1–9, and 1.0, modifying ***x*** by steps Δ***x*** equal to 0.1. The DFs of eq 6 and eq 7b have been evaluated for α = 0.57 (***x*** = −0.43), α = 0.70 (***x*** = −0.30), and α = 0.85 (***x*** = −0.15). For each system, the best value of ***x*** was defined for the DFs given by eqs 1–7a as described in more detail below. If for the resulting ***x*** we have 0.0 ≤ ***x*** ≤ 1.0 for a DF defined by one of the eq 1–5 values, the interpolation was successful and the DF expression can be used for the system considered. Similarly, if for the resulting ***x*** we have −0.43 ≤ ***x*** ≤ 1.0 for a DF defined by one of the eqs 6 or 7 the interpolation was successful and the DF expression can be used for the system considered. Otherwise, extrapolation was used, and the corresponding generic DF was found not to be able to describe the system successfully.

Before closing this section, it is worthwhile to compare the limiting forms of the DFs tested here by inspecting Figure 1 and considering their Taylor expansions. As can be seen, over the range of ***s*** considered the exchange enhancement factor of the RPBE DF is consistently larger than that of the PBE DF, which is consistently larger than those of the PBEsol and PBEα = 0.57 DFs. As larger ***s*** values tend to correspond to higher barriers,^98^ we may expect the RPBE barriers to be consistently higher than the PBE barriers, in agreement with conventional wisdom.^7^ Furthermore, replacing PBE with PBEα or PBEsol should, according to Figure 1, be successful in increasing the tunability of SRP-DFs. Finally, as the exchange enhancement factor of the PBEsol DF is lower than that for PBEα = 0.57 for ***s*** up to about 4, one would expect the use of this DF as the lower limit of the SRP-DF to be most successful at increasing its tunability. Finally, one observes that at low ***s*** values the RPBE, PBE, and PBEα = 0.57 DFs all behave similarly, while the exchange enhancement factor of the PBEsol DF is clearly lower for small ***s***-values. The enhancement factors of the RPBE, PBE, and PBEα = 0.57 DFs start to diverge only for values of ***s*** greater than 1. To understand this, it is useful to consider the Taylor expansions of the four DFs up to the fourth order in ***s***.
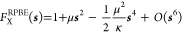
12a
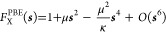
12b
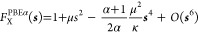
12c
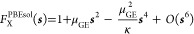
12d

The Taylor expansions help us to understand much of the observed behavior. For instance, at low ***s*** values the exchange-enhancement factors of the RPBE, PBE, and PBEα = 0.57 DFs are similar because their Taylor expansions are identical up to second order in ***s***. At these small values of ***s*** the exchange-enhancement factor of the PBEsol DF is significantly smaller, and it remains smaller up to ***s*** ≈ 4 because it uses the gradient expansion form of μ (μ_GE_ = 0.1235) that is accurate for slowly varying electron gases.^87^ As a result, the exchange-enhancement factor ends up being much smaller at small ***s*** even though the form of its second-order Taylor expansion is identical to that of the others, which however all use μ = 0.21951. At larger ***s***, where the ***s***^4^ term kicks in, differences between RPBE, PBE, and PBEα may be understood from the different coefficients in front of the term μ^2^***s***^4^/κ, which equal −0.5, −1, and −1.377 for RPBE, PBE, and PBEα = 0.57, respectively. As noted before, differences between the exchange enhancement factors that occur for large values of the reduced density gradient, as observed in Figure 1, may be relevant to the calculation of the exchange energy in regions where ***s*** is large and the density is low.^90,94^ Finally, we note that in Figure 1 the exchange-enhancement factors all obey the condition that *F*_X_(***s***) ≤ 1.804, which is known as a local Lieb-Oxford bound and which is a sufficient condition^36,69^ for the global Lieb-Oxford bound on the exchange energy^88^ being obeyed. As discussed by Marques and co-workers, this is not a necessary condition, and in real systems the local Lieb-Oxford bound may be violated.^99^

### 2C Computational Details

The minimum barrier height is computed as follows.
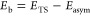
13

In eq 13*E*_TS_ is the energy of the system (molecule + surface) at the minimum barrier geometry, while *E*_asym_ is the energy of the system with the molecule in its equilibrium geometry at a distance from the surface such that molecule and surface no longer interact. In the so-called medium algorithm that we use, which is defined and explained in detail in ref (8), the surface is set up following DFT geometry optimizations of the bulk lattice (to determine the bulk lattice constant(s) with the DF used) and of the metal slab representing the surface (to determine interlayer spacings in the metal surface slab exposed to vacuum according to the DF used). The geometry of the molecule relative to the surface is taken from earlier SRP-DFT calculations as described in ref (8) (see also Table 2 of ref (8)). In the asymptotic geometry the equilibrium distance of the molecule is likewise computed with the DF tested.^8^ A crucial point is that the surface is not allowed to relax with respect to the incoming molecule in the calculation of *E*_TS_. A minor difference with ref (8) is that in the present work the geometry optimization of the bulk representing the surface was done using the geometry optimization method implemented in VASP. In the earlier calculations of ref (8), a parabola was fitted to the energy of the bulk as a function of the lattice constant, and minimization was used to establish the bulk lattice constant. The new approach led to small differences in the values of the barrier heights (of 10 meV or less) with respect to the early results when they were available for the particular DF tested.

All DFT calculations were performed with a user-modified version of the Vienna ab initio simulation package^100−103^ (VASP5.4.4). We also used the Atomic Simulation Environment (ASE)^104,105^ as a convenient interface package. All calculations using the vdW-DF1 or vdW-DF2 C functionals were done with the algorithm of Román-Pérez and Soler^106^ to speed up their evaluation. All other details regarding the calculations (concerning the pseudopotentials used, the handling of spin-polarization in systems containing Ni, the number of metal layers in the slab representing the surface, the size of the surface unit cell, etc.) are the same as in ref (8), to which we refer for these details.

## 3 Results and Discussion

### 3A Equilibrium Lattice Constants Computed with Mixed Density Functionals

Equilibrium lattice constants computed with the mixed density functional expressions not incorporating the van der Waals interaction are shown in Table S1 of the Supporting Information, stepping through ***x*** in SRP***x*** and SRP***x***sol in steps of 0.1 (results for the other mixed DFs not shown). Comparing with zero-point energy-corrected experimental values we obtain the usual result that the PBE DF somewhat underestimates and that RPBE overestimates lattice constants.^107,108^ The PBEsolc DF (we recall that PBEsolc is the name we use for the DF with PBEsol exchange and PBE correlation) tends to somewhat underestimate the lattice constant. The PBEsol DF would be expected to do rather well for the lattice constant,^108^ and we suspect that PBEsolc somewhat underperforms as using PBE correlation with PBEsol exchange should lead to a somewhat unbalanced functional.^87^ One might of course vary ***x*** in the SRP***x***sol DF to obtain the correct lattice constant, but this is not likely to lead to the correct barrier height, as GGA DFs yielding good molecule–surface interaction energies tend to overestimate metal lattice constants.^87,109^

### 3B Performance of Limiting Forms of the Mixed Density Functionals

**Figure 2:**
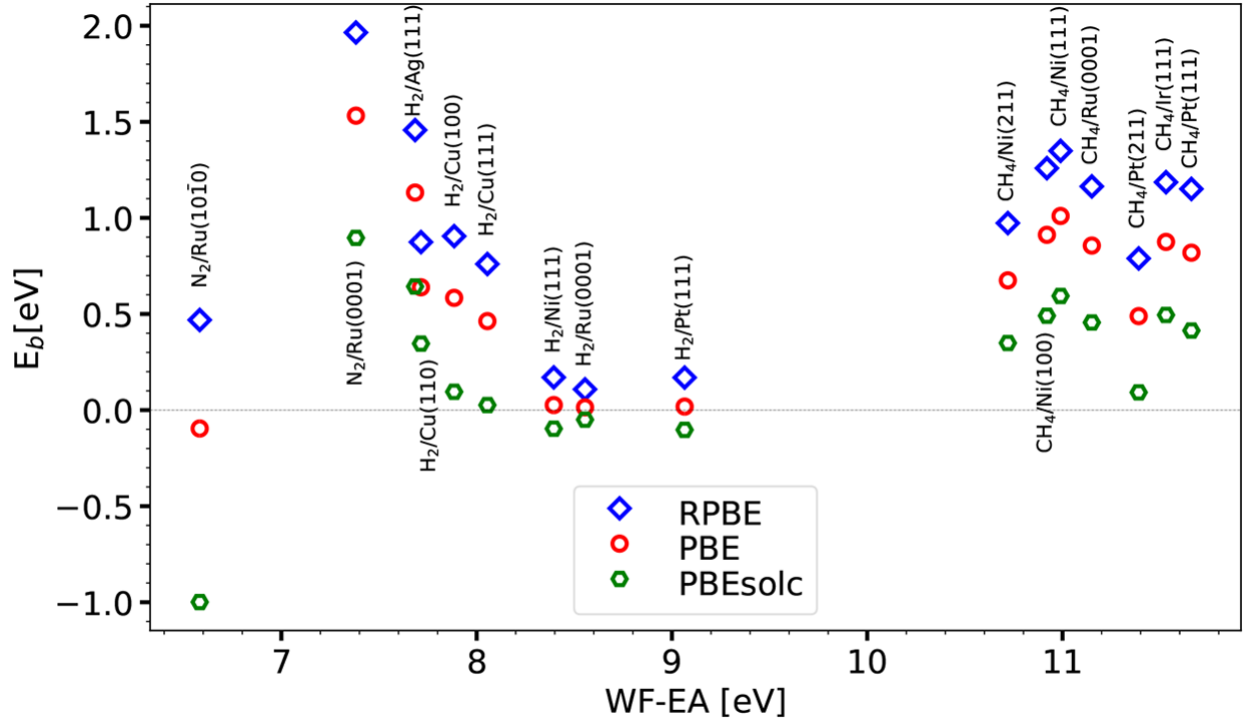
Barrier heights *E*_b_ computed with the PBEsolc, PBE, and RPBE DFs are shown as a function of the charge transfer parameter for the 16 systems present in the SBH16 database.

To get an impression of how the mixed density functional expressions will perform as generic expressions for fitting SRP functionals, it is a good idea to look at how their limiting forms perform and compare. For this, we first consider the limiting forms of the mixed expressions not using van der Waals correlation functionals, i.e., SRP***x*** (eq 1) and SRP***x***sol (eq 2), which are PBE and RPBE, and PBEsolc (we recall that this is the name we use for the DF with PBEsol exchange and PBE correlation) and RPBE. Figure 2 shows that for each system in the SBH16 database the barrier height obtained with PBE is lower than that obtained with RPBE, which correlates well with the finding that PBE often underestimates while RPBE often overestimates barrier heights.^7^ Our results show for all systems investigated here that using RPBE instead of PBE raises the barrier height because the energy of the transition state increases more than the energy of the reactants (of the system with the molecule in the gas phase). This is not completely trivial as the same change in barrier height may also result from the energy of the transition state decreasing less than the energy of the reactants.^98^ Also, for each system in the SBH16 database, the barrier height obtained with PBEsolc is lower than that obtained with PBE, suggesting that for the purpose of fitting barrier heights, the SRP***x***sol expression will be tunable over a wider range than the SRP***x*** expression. The barrier heights computed with the PBEsolc, PBE, and RPBE functionals may also be found in Table S2 of Supporting Information.

**Figure 3:**
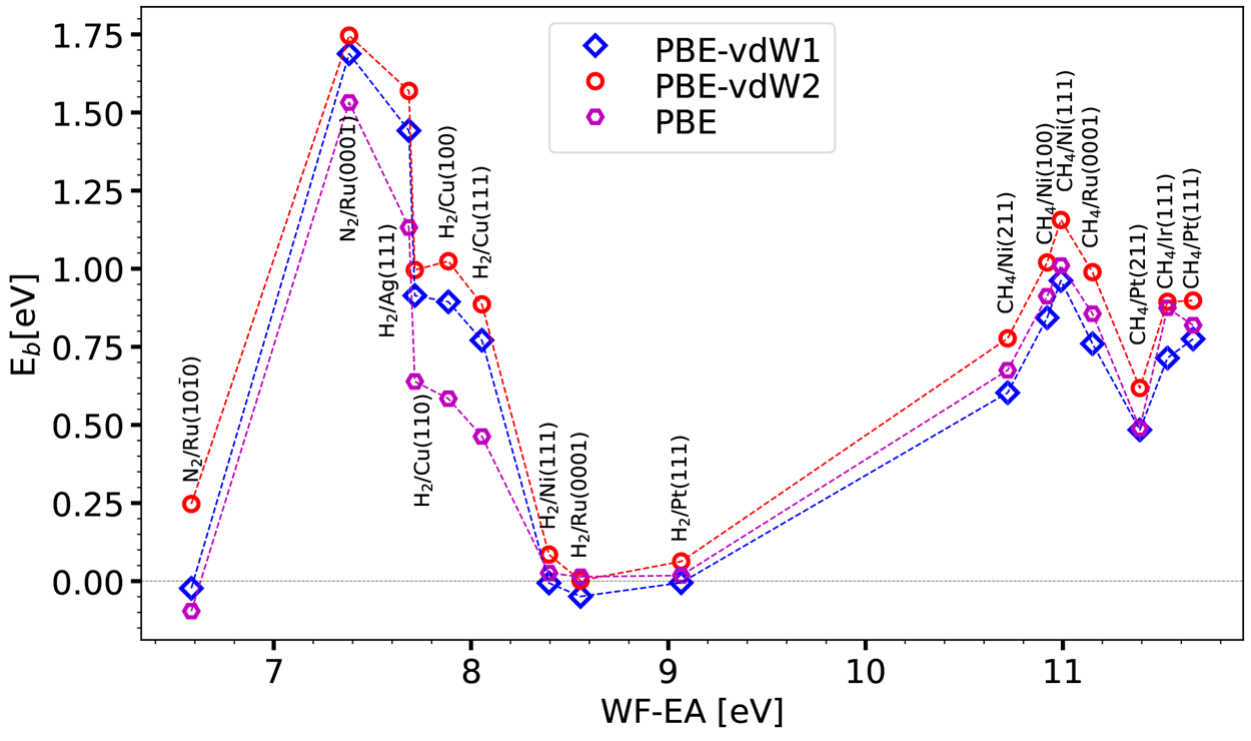
Barrier heights *E*_b_ computed with the PBE, the PBE-vdW1, and the PBE-vdW2 DFs are shown as functions of the charge transfer parameter for the 16 systems present in the SBH16 database.

**Figure 4:**
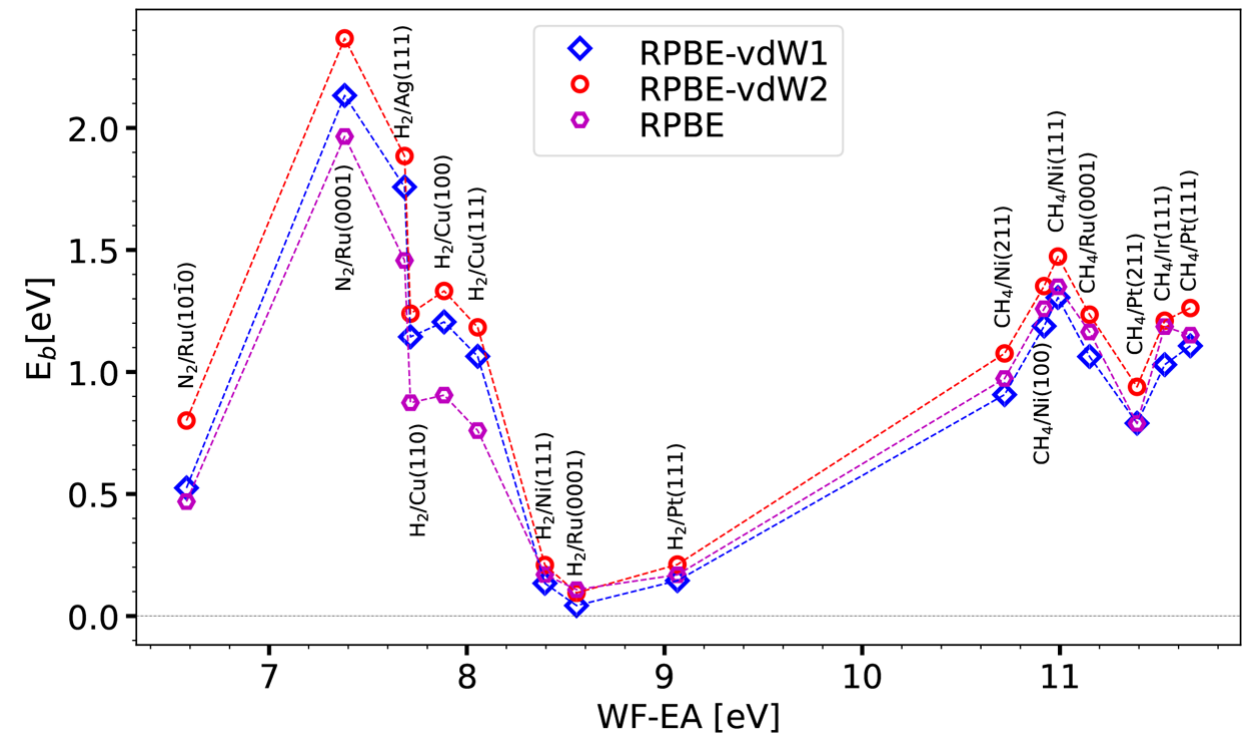
Barrier heights *E*_b_ computed with the RPBE, the RPBE-vdW1, and the RPBE-vdW2 DFs are shown as a function of the charge transfer parameter for the 16 systems present in the SBH16 database.

Barrier heights obtained for each system in the SBH16 database with the limiting forms of the SRP***x*** (eq 1), SRP***x***vdW1 (eq 3), and SRP***x***-vdW2 (eq 4) expressions are shown in Figure 3 for PBE, PBE-vdW1, and PBE-vdW2 and in Figure 4 for RPBE, RPBE-vdW1, and RPBE-vdW2. Whether PBE or PBE-vdW1 yields the lowest barrier height is seen to depend on the value of Δ*E*_CT_: for Δ*E*_CT_ ≤ 8.055 eV, PBE yields the lowest barrier height, while for Δ*E*_CT_ ≥ 8.395 eV, PBE-vdW1 yields the lowest barrier height. While this might look odd, one should remember that the correlation part of the vdW-DF1 functional is not just a van der Waals term that is added to an energy expression excluding the attractive dispersion interaction (e.g., the PBE energy). Rather, the vdW-DF1 correlation functional is a different correlation functional from the PBE correlation functional. There is thus no a priori reason that the PBE-vdW1 energy should always be lower than the PBE energy or vice versa. Furthermore, the barrier obtained with PBE-vdW2 is almost always higher than that obtained with both PBE-vdW1 and PBE (only for H_2_ + Ru(0001) is the barrier higher for PBE-vdW1 than for PBE-vdW2). The findings for RPBE, RPBE-vdW1, and RPBE-vdW2 (Figure 4) are analogous to those for PBE, PBE-vdW1, and PBE-vdW2 (Figure 3). The barrier heights computed with the PBE, PBE-vdW1, PBE-vdW2, and RPBE functionals may be found in Table S2 of the Supporting Information, and the barrier heights computed with the RPBE-vdW1 and RPBE-vdW2 functionals may be found in Table S3 of the Supporting Information.

**Figure 5:**
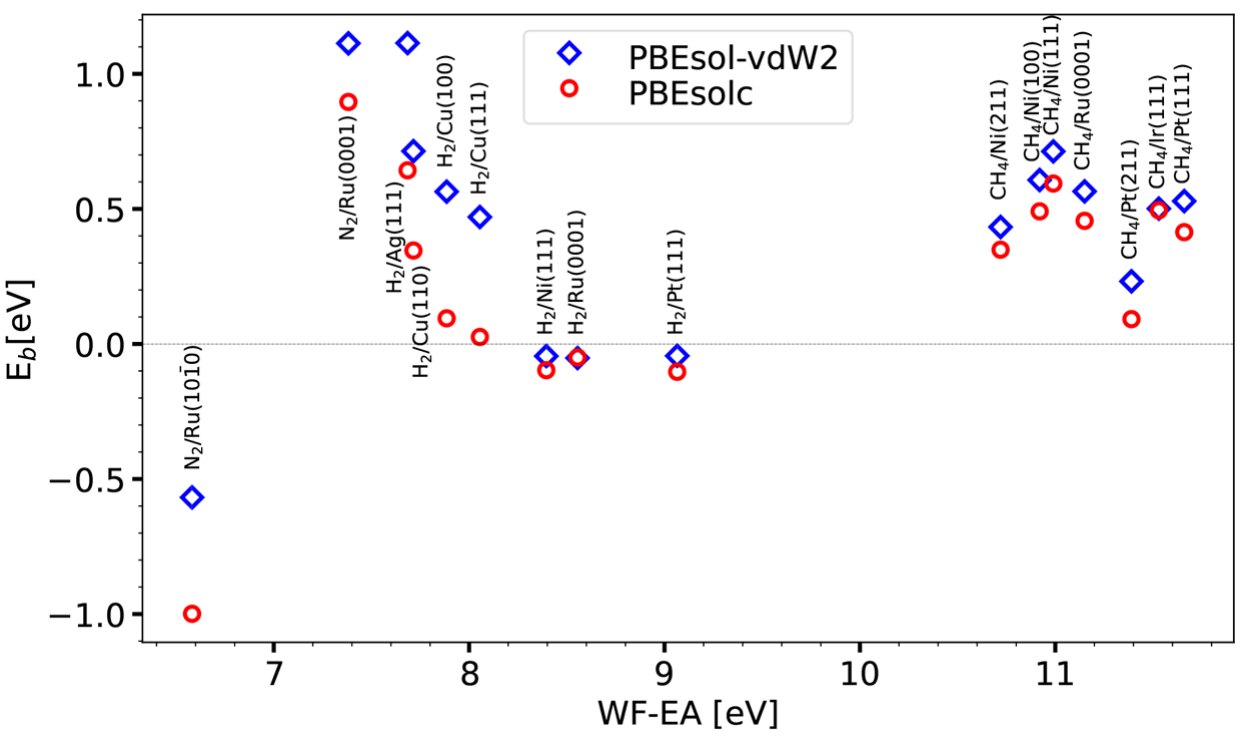
Barrier heights *E*_b_ computed with the PBEsolc and PBEsol-vdW2 DFs are shown as a function of the charge transfer parameter for the 16 systems present in the SBH16 database.

Barrier heights obtained for each system in the SBH16 database with the lower-limit forms of the SRP***x***sol (eq 2) and SRP***x***sol-vdW2 (eq 5) expressions are shown in Figure 5 for PBEsolc and PBEsol-vdW2. As can be seen, the barriers obtained with PBEsolc-vdW2 are always higher than those obtained with PBEsolc, suggesting that the SRP***x***sol-vdW2 DF may be slightly less tunable than the SRP***x***sol DF, which yields very low barriers. The barrier heights computed with the PBEsolc functional are listed in Table S2 of the Supporting Information, and the barrier heights computed with the PBEsol-vdW2 functional are listed in Table S3 of the Supporting Information.

Finally, barrier heights obtained with the PBEα-vdW1 and PBEα-vdW2 DFs are compared in Figure S1 of the Supporting Information for α = 0.57, which is the lowest value of α used here. Figure S1 shows that the PBEα-vdW1 DF consistently yields barrier heights lower than those of the PBEα-vdW2 DF with α = 0.57. This suggests that the PBEα-vdW1 DF is a better tunable mixed DF than the PBEα-vdW2 DF, as the RPBE-vdW1 and RPBE-vdW2 DFs overestimate the barrier height for each system in the SBH16 database (see the discussion of Table 1 below).

**Table 1:** Performance of the DFs That Represent Limiting Forms of the Mixed Density Functionals Tested on the SBH16 Database Using the Medium Algorithm^a^

	Med Algo			
Functional	MAE	MSE	MAE-SBH17	MSE-SBH17
PBE	0.107	–0.065	0.103	–0.058
RPBE	0.235	0.235	0.228	0.228
PBEsolc	0.458	–0.458		
PBEsol-vdW-DF2	0.269	–0.265		
PBE-vdW-DF1	0.128	–0.020		
PBE-vdW-DF2	0.148	0.117	0.141	0.112
PBEα+57-vdW-DF1	0.209	–0.185		
PBEα57-vdW-DF2	0.132	–0.042	0.124	–0.040
RPBE-vdW-DF1	0.278	0.278		
RPBE-vdW-DF2	0.424	0.424		
Average	0.239	0.002		

a The mean absolute errors (MAEs) and mean signed errors (MSEs) are presented in eV for all density functionals investigated here. For the density functionals for which these results are available we also present MAEs and MSEs computed previously for the closely related SBH17 database.^8^.

Table 1 shows mean absolute errors (MAEs) and mean signed errors (MSEs) for the SBH16 database, also comparing to the previous SBH17 results for those DFs that have previously been tested on this database.^8^ Here the error for a specific system is defined as the difference between the barrier height computed here and the reference value tabulated in ref (8) for that system. As can be seen, the MAEs and MSEs computed here for SBH16 differ from previous results known from SBH17 by no more than 10 meV, underscoring the reliability of the results presented here. As previously found, the PBE DF is the best-performing DF in terms of the MAE, the MAE being lowest for the PBE DF. Importantly for this study, the DFs serving as upper limits for mixed DFs here (RPBE for SRP***x*** of eq 1 and SRP***x***sol of eq 2, RPBE-vdW1 for SRP***x***-vdW1 of eq 3 and for SRP***x***-vdW1-ext of eq 6a, and RPBE-vdW2 for SRP***x***vdW2 of eq 4, SRP***x***sol-vdW2 of eq 5, and SRP***x***-vdW2-ext of eq 7a) all have their MSEs equal to their MAEs, suggesting that these DFs all systematically overestimate the barrier height. This is actually a good quality of a functional that is meant to serve as the upper-limit form of a mixed DF. The PBEsolc DF, which is the lower-limit form of the SRP***x***sol DF of eq 2, shows an MSE that is equal to minus its MAE, suggesting that this DF systematically underestimates the barrier height. This is a good quality of a functional that is meant to serve as the lower-limit form of a mixed DF, and in view of the behavior of the RPBE DF we expect that the SRP***x***sol DF of eq 2 will perform well as a generic expression for reproducing barrier heights by tuning its ***x***-parameter. Unfortunately PBE (the lower-limit-form of SRP***x*** of eq 1), PBE-vdW1 (the lower limit of SRP***x***-vdW1 of eq 3), PBE-vdW2 (the lower limit of SRP***x***-vdW2 of eq 4), PBEsol-vdW2 (the lower limit of SRP***x***solvdW2 of eq 5), PBEα57-vdW1 (the lower limit of SRP***x***-vdW1-ext of eq 6a), and PBEα57-vdW2 (the lower limit of SRP***x***-vdW2-ext of eq 7a) all have the idea that their MSE is not equal to minus their MAE, meaning that these DFs do not systematically underestimate the barrier height for the systems in SBH17. Of these DFs, on the basis of the correspondence between their MAE and the negative of their MSE, PBEsol-vdW2 and PBEα57-vdW1 are expected to function best as lower-limit forms, and consequently the mixed DFs SRP***x***sol-vdW2 and SRP***x***-vdW1-ext are also expected to perform well as tunable mixed DFs.

### 3C Performance of Mixed Density Functionals as Tunable SRP DFs

**Figure 6:**
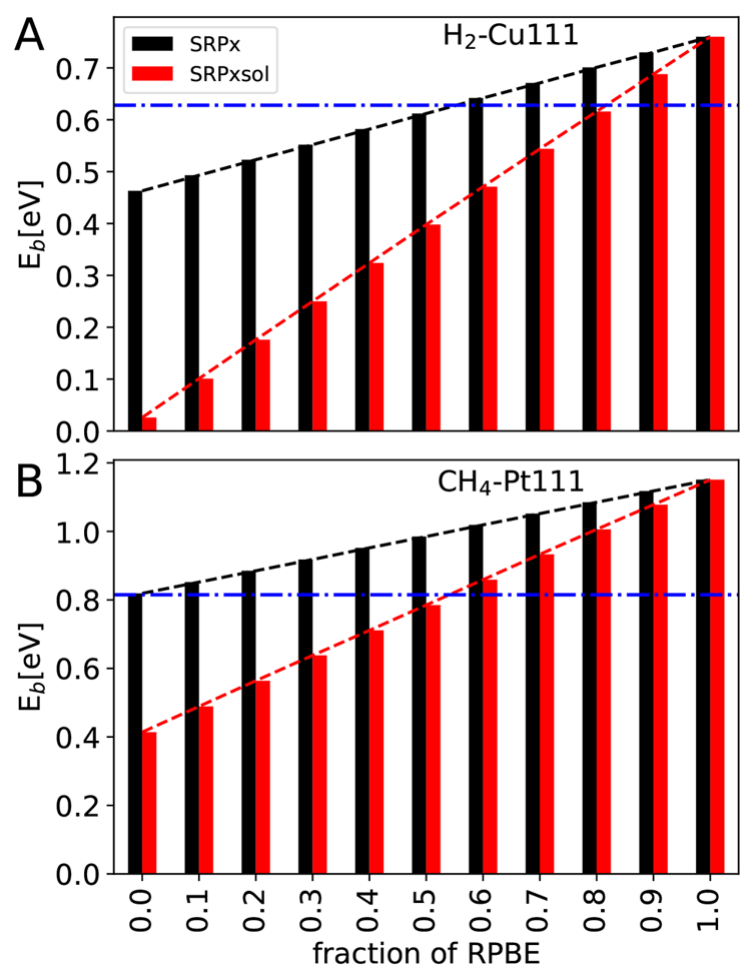
Barrier heights computed with the SRP***x*** DF (black bars) and the SRP***x***sol DF (red bars) are shown as a function of the fraction of RPBE exchange ***x***, for (A) H_2_ + Cu(111) (upper panel) and (B) CH_4_ + Pt(111) (lower panel). Blue horizontal lines indicate the reference value of the barrier height for these systems.^8^ The black and red dashed lines linearly interpolate the barrier height as a function of ***x*** for SRP***x*** and SRP***x***sol DFs, respectively. The optimal value of ***x*** is equal to the value of ***x*** for which these lines intersect with the blue lines.

Figure 6 illustrates how we find the optimal value of ***x*** for each mixed DF by showing how this was done for the particular examples of the H_2_ + Cu(111) and CH_4_ + Pt(111) systems using the mixed DFs SRP***x*** and SRP***x***sol of eqs 1 and 2. As Figure 6A,B shows, the barrier height obtained with a mixed DF typically depends linearly on ***x***. This means that the optimal value of ***x*** can be found using linear interpolation, i.e., from the point where the linearly interpolated barrier height curves (the sloping red and black lines) intersect the horizontal blue line, representing the reference value of the barrier height. If ***x*** does not fall between the limits of the mixed DF (0 and 1 for the expressions of eqs 1–5, and −0.43 and 1 for eqs 6 and 7) a value of ***x*** can be found by extrapolation. We have not tested whether the DFs that may be obtained by extrapolation lead to reasonable values of the minimum barrier height; we do not recommend their use. However, the values of ***x*** obtained in this way may be used in the calculation of the correlation coefficients discussed in the next Section.

**Figure 7:**
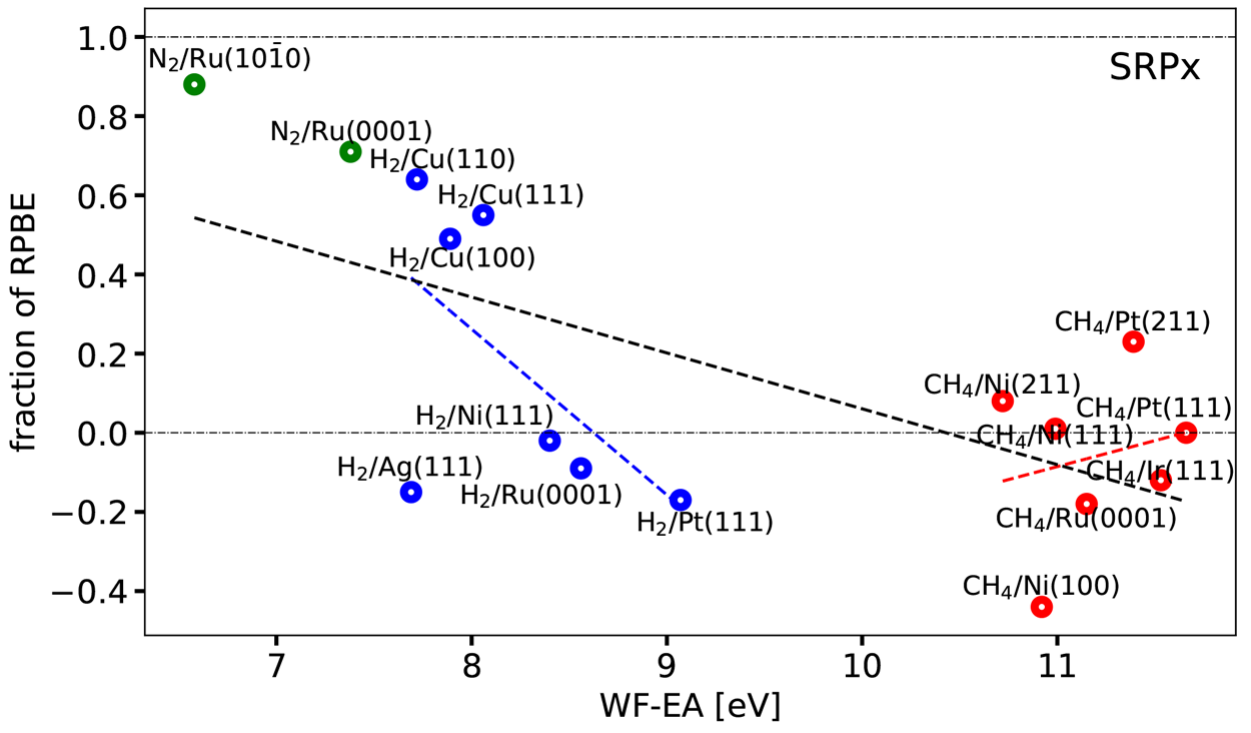
Optimum fraction of RPBE exchange ***x*** is shown as a function of Δ*E*_CT_ for the SRP***x*** DF (eq 1). Values falling between the two horizontal dot-dashed black lines could be obtained by the interpolation procedure illustrated in Figure 6. The green, blue, and red symbols correspond to N_2_, H_2_, and CH_4_ + metal surface systems, respectively. The black, blue, and red dashed lines provide the linear fits corresponding to the Pearson correlation coefficients computed for all molecules, H_2_, and CH_4_ + metal surface systems, respectively, without omitting systems.

**Figure 8:**
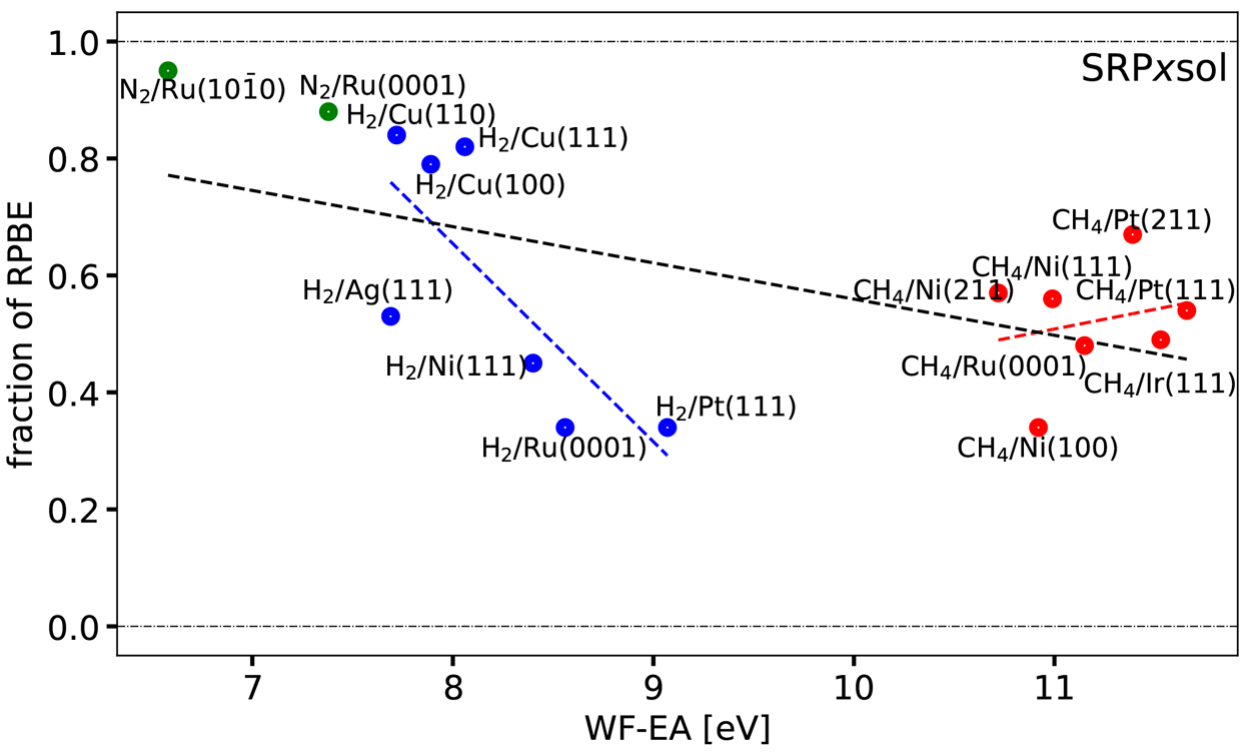
Optimum fraction of RPBE exchange ***x*** is shown as a function of Δ*E*_CT_ for the SRP***x***sol DF (eq 2). Values falling between the two horizontal dot-dashed black lines could be obtained by the interpolation procedure illustrated in Figure 6. The green, blue, and red symbols correspond to N_2_, H_2_, and CH_4_ + metal surface systems, respectively. The black, blue, and red dashed lines provide the linear fits corresponding to the Pearson correlation coefficients computed for all molecules, H_2_, and CH_4_ + metal surface systems, respectively, without omitting systems.

Figures 7 and 8 show the optimal ***x*** coefficients computed for the SRP***x*** and SRP***x***sol DFs of eqs 1 and 2, respectively, as a function of Δ*E*_CT_. These coefficients are also listed for each DF in Table S4. Figure 7 shows that obtaining the optimum value of ***x*** for the SRP***x*** DF required extrapolation to negative values for several H_2_-metal and CH_4_-metal surface systems. The use of this mixed DF is therefore not guaranteed to yield a useful SRP DF for systems like the ones investigated here. From the point of view of tunability, the opposite is true for the SRP***x***sol DF, for which we obtained a value of ***x*** falling between 0 and 1 for all systems in the SBH16 database (see Figures 7 & 8).

**Figure 9:**
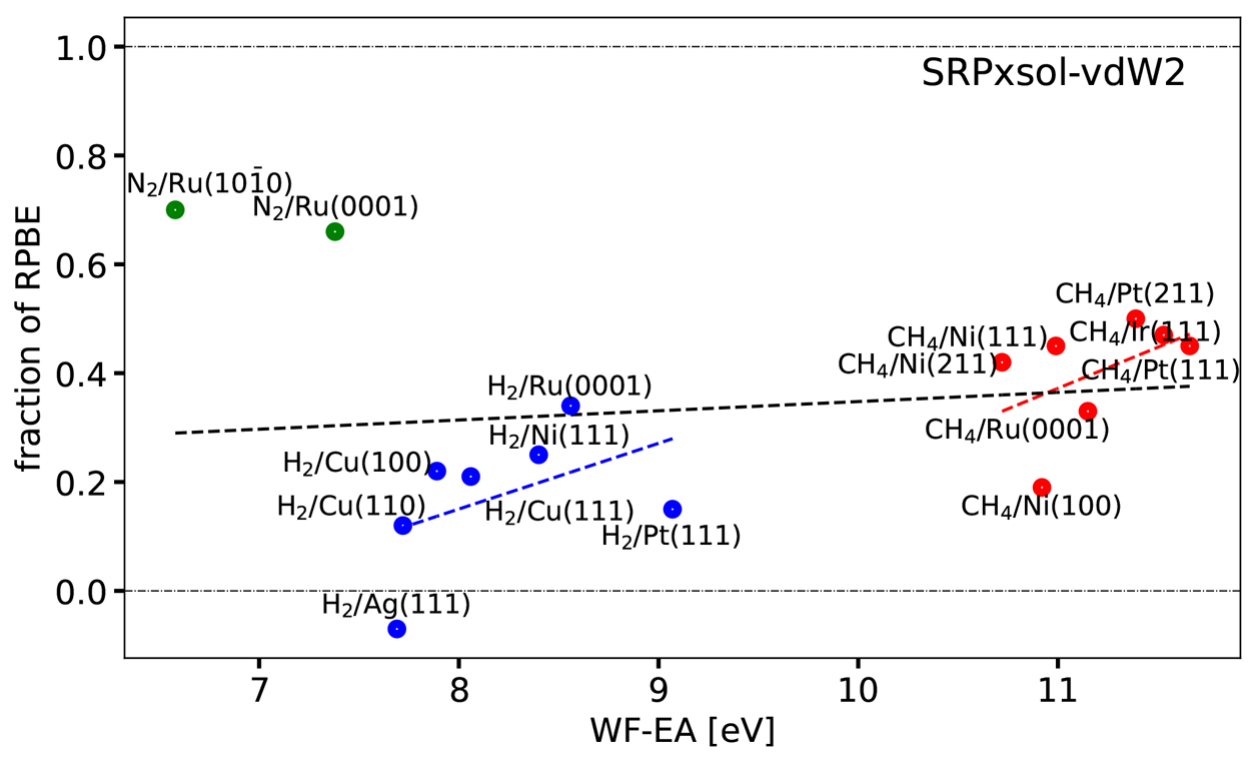
Optimum fraction of RPBE exchange ***x*** is shown as a function of Δ*E*_CT_ for the SRP***x***sol-vdW2 DF (eq 5). Values falling between the two horizontal dot-dashed black lines could be obtained by the interpolation procedure illustrated in Figure 6. The green, blue, and red symbols correspond to N_2_, H_2_, and CH_4_ + metal surface systems, respectively. The black, blue, and red dashed lines provide the linear fits corresponding to the Pearson correlation coefficients computed for all molecules, H_2_, and CH_4_ + metal surface systems, respectively, without omitting systems.

Figure 9 shows the optimal ***x*** coefficients computed for the SRP***x***sol-vdW2 DF of eq 5 as a function of Δ*E*_CT_. These coefficients are also listed for this DF in Table S5. Figure 9 shows that obtaining the optimum value of ***x*** for the SRP***x***sol-vdW2 DF only required extrapolation to a negative value for H_2_ + Ag(111). This system was classified as problematic in the SBH17 study, with all DFs tested there yielding large MAEs for this system.^8^ While we conclude that the use of this mixed DF is not guaranteed to yield a useful SRP DF for systems like the ones investigated here, we find that it performs rather well and that it can probably be used if an SRP DF is desired with vdW-DF2 correlation in it. Note that, when coupled to their original partner exchange functionals,^31,32^ the vdW-DF2 functional^32^ yields a better description of the S22 database binding energies of gas-phase dimers (MAE of 22 meV)^32^ than the vdW-DF1 functional^32^ (MAE of 41 meV).^31^ However, the vdW-DF1 functional^31^ generally yields a better description of bulk solids^110^ than the vdW-DF2 functional.^32^ The greater tunability of the SRPxsol DF comes from using an exchange enhancement factor that is more appropriate for solids and surfaces than for molecules through the use of μ_GE_ = 0.1235 in PBEsol^87^ instead of μ = 0.21951 in PBE.^69^ This leads to overall lower values of the gradient enhancement factor as a function of ***s***, which leads to lower barriers.^98^

**Figure 10:**
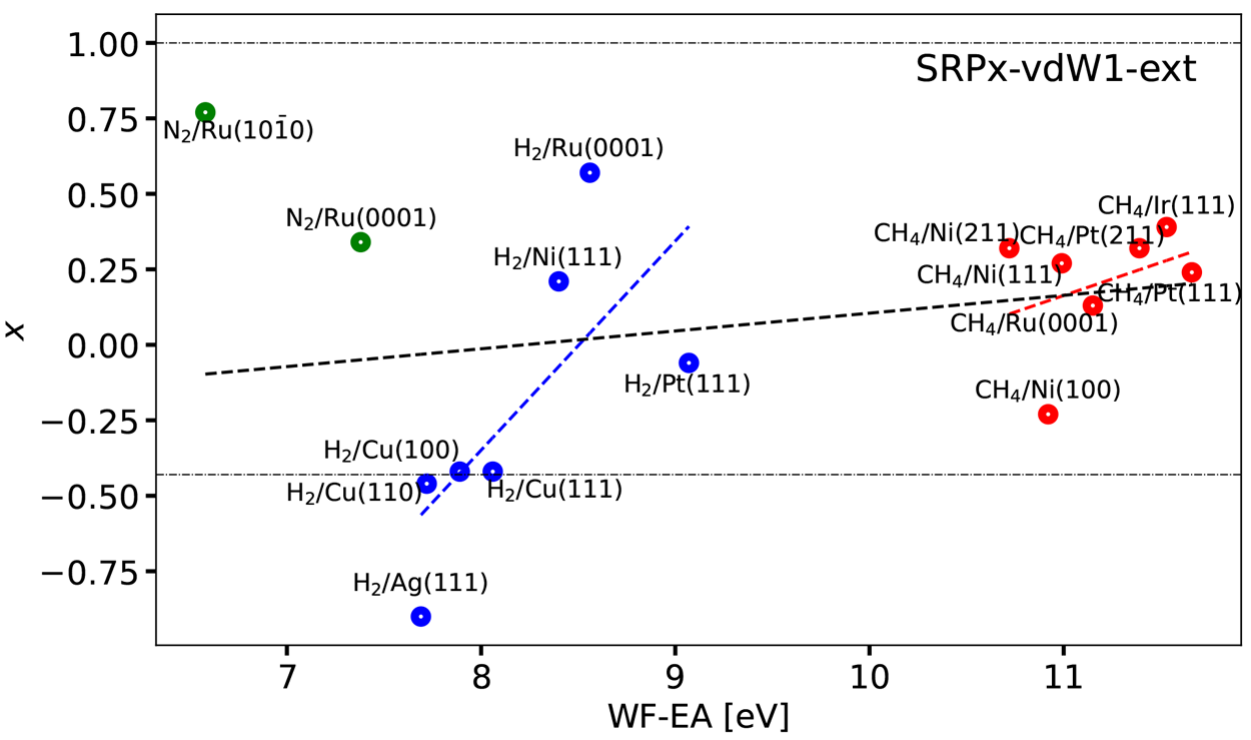
Optimum mixing parameter ***x*** is shown as a function of Δ*E*_CT_ for the SRP***x***-vdW1-ext DF (eq 6a). Values falling between the two horizontal dot-dashed black lines could be obtained by the interpolation procedure illustrated in Figure 6. The green, blue, and red symbols correspond to N_2_, H_2_, and CH_4_ + metal surface systems, respectively. The black, blue, and red dashed lines provide the linear fits corresponding to the Pearson correlation coefficients computed for all molecules, H_2_, and CH_4_ + metal surface systems, respectively, without omitting systems.

Figure 10 shows the optimal ***x*** coefficients computed for the SRP***x***vdW1-ext DF of eq 6a as a function of Δ*E*_CT_. These coefficients are also listed in Table S5. Figure 10 shows that obtaining the optimum value of ***x*** for the SRP***x***-vdW1-ext DF only required extrapolation to a negative value for H_2_ + Cu(110) and H_2_ + Ag(111). The latter system was classified as problematic in the SBH17 study, with all DFs tested there yielding large MAEs for this system.^8^ The use of the SRP***x***-vdW1-ext mixed DF is not guaranteed to yield a useful SRP DF for systems like the ones investigated here, but we find that it performs rather well just like SRP***x***sol-vdW-DF2, and SRP***x***-vdW1-ext can be used if an SRP-DF is desired with vdW-DF1 correlation in it. As noted above, when partnered with their original exchange functionals vdW-DF1 yields better descriptions of bulk solids, while vdW-DF2 tends to be better for binding energies of gas-phase dimers. Finally, we note that the H_2_ + Ag(111) system is among the 1 (2) systems for which the optimum fraction of RPBE exchange could not be obtained through interpolation with the SRP***x***sol-vdW2 DF (the SRP***x***-vdW1 DF), as can be seen from Figure 9, respectively. This confirms the analysis of ref (8), which already suggested revisiting this system with new experiments and calculations.

Figures S2, S3, and S4 show the optimal ***x*** coefficients computed for the SRP***x***-vdW1, SRP***x***-vdW2, and SRP***x***-vdW2-ext DFs of eqs 3, 4, and 7a, respectively, as a function of Δ*E*_CT_. These coefficients are also listed for each DF in Tables S4 and S5. Figures S2–S4 show that obtaining the optimum value of ***x*** for these three mixed DFs required extrapolation to negative values for several H_2_-metal surface and in most cases also for several CH_4_-metal surface systems, with SRP***x***-vdW2 performing particularly poorly. The above suggests that these three mixed DFs, and especially SRP***x***-vdW2, should perhaps not be the first choice for deriving a new SRP-DF for a system like those present in the SBH16 database.

We end by noting that the ability of the SRP***x***-vdW2 (Figure S3), SRP***x***-vdW2-ext (Figure S4), and SRP***x***sol-vdW2 (Figure 9) to interpolate ***x*** increases in the order SRP***x***-vdW2 < SRP***x***-vdW2-ext < SRP***x***sol-vdW2. This is related to the tunability of the SRP***x***sol-vdW2 DF being greatest from the use of μ_GE_ = 0.1235 in PBEsol,^87^ which affects the gradient enhancement factor already to second order in ***s*** (see Figure 1). Through the use of PBEα the SRP***x***-vdW2-ext DF also has a smaller gradient enhancement factor that is smaller than that of SRP***x***-vdW2 (which uses PBE), but here the decrease comes only from the fourth-order dependence of ***s***, and as Figure 1 shows the gradient enhancement factor of SRP***x***-vdW2-ext is intermediate between that of PBE and PBEsol. As noted before, the lower the gradient enhancement factor of a DF is as a function of ***s***, the lower the barriers the DF will produce,^98^ which explains the extent of the tunability of the three mixed DFs using vdW2 correlation that were tested here.

### 3D Correlation of the Mixing Parameter with the Charge Transfer Parameter

Table 2 shows correlation coefficients (or Pearson product-moment correlation coefficients)^111^*r*_*xy*_ describing the correlation between the charge transfer parameter taken as independent variable and the mixing coefficient ***x*** taken as dependent variable, for the seven mixed DFs tested here. Including all systems, the *r*_*xy*_ values are clearly negative for the SRP***x*** and the SRP***x***sol DFs. The same is true for these DFs if only the H_2_-metal systems are considered, and for these systems the *r*_*xy*_ values get close to the value of −1 indicating a nearly perfect linear relationship if the H_2_ + Ag(111) system, for which the reference barrier height is somewhat suspect, is not considered. For CH_4_-metal systems the values of *r*_*xy*_ only take on negative values if the CH_4_ + Ru(0001) and Ni(100) systems, for which the reference barrier heights are also somewhat suspect, are not considered, and these values are small in absolute value. These findings support the analysis of ref (8), which called for more accurate reference data for H_2_ + Ag(111), CH_4_ + Ni(100), and CH_4_ + Ru(0001).

**Table 2:** Correlation Coefficients Computed for the Dependence of the Optimum Fraction of RPBE Exchange ***x*** on the Charge Transfer Parameter for the Mixed DFs Tested^a^

type SRP	All-16	All-12	7 H_2_-metal	6 H_2_-metal	7 CH_4_-metal	5 CH_4_-metal
SRP***x***	–0.648	–0.617	–0.584	–0.927	0.211	–0.239
SRP***x***sol	–0.543	–0.409	–0.761	–0.91	0.228	–0.174
SRP***x***-vdW-DF1	0.264	0.528	0.752	0.684	0.362	0.003
SRP***x***-vdW-DF2	0.205	0.483	0.751	0.695	0.500	0.801
SRP***x***sol-vdW-DF2	0.147	0.447	0.473	0.209	0.483	0.609
SRP***x***-vdW-DF1-ext	0.236	0.521	0.716	0.627	0.361	0.003
SRP***x***-vdW-DF2-ext	0.148	0.423	0.741	0.671	0.420	0.428

a Computed correlation coefficients are provided for the 7 H_2_-metal surface systems present in the SBH16 database, the 6 H_2_-metal surface systems obtained once H_2_ + Ag(111) is removed, the 7 CH_4_-metal surface systems in the SBH16 database, the 5 CH_4_-metal surface systems that remain after CH_4_ + Ru(0001) and Ni(100) are removed, the 16 systems (All-16) present in the SBH16 database, and the 12 systems (All-12) that remain after the 3 systems already mentioned and N_2_ + Ru(101̅0) are removed.

The finding of negative correlation coefficients as observed here for the SRP***x*** and SRP***x***sol DFs is what we expected to see for several reasons. First of all, the MAE of the RPBE DF was previously found to increase from 88 to 167 to 336 meV going from N_2_-metal systems to H_2_-metal systems to CH_4_-metal systems,^8^ respectively, i.e., going from small values of the charge transfer parameter to large values (see, e.g., Table S2 and Figure 7 for how the charge transfer parameter varies with the type of system). The opposite is true for the PBE DF, where the MAE was found to decrease from 409 to 80 to 45 meV going from N_2_-metal systems to H_2_-metal systems to CH_4_-metal systems,^8^ respectively. Second, tests on several systems suggest that for systems characterized by charge transfer parameters less than 7 eV even RPBE exchange is not repulsive enough to avoid underestimating the barrier height.^35^ However, it is also clear that when all three types of systems are considered, the correlation is not that strong, suggesting that when a mixed functional with a fraction of PBE correlation is used, the optimum mixing coefficient also depends on other properties of the system than the charge transfer parameter. In this context we note that *r*_*xy*_ for all systems decreases in absolute value if the four systems with suspect reference values (N_2_ + Ru(101̅0), CH_4_ + Ru(0001), CH_4_ + Ni(100), and H_2_ + Ag(111))^8^ are excluded from the SBH16 database (see Table 2), which would not be expected if ***x*** would only depend on the charge transfer parameter and the relationship would be linear.

The computed values of the correlation coefficients for the DFs incorporating van der Waals correlation are rather different from the values calculated for SRP***x*** and SRP***x***sol, which incorporate the PBE correlation. Restricting ourselves to the mixed DFs that exhibit high tunability, i.e., SRP***x***sol-vdW2 and SRP***x***-vdW1-ext, we see that the former one only exhibits positive correlation coefficients and that the latter one exhibits correlation coefficients that are either positive or close to zero. The reasons for the different values of the correlation coefficients of SRP***x*** and SRP***x***sol on the one hand (mostly negative) and the other DFs incorporating van der Waals correlation on the other hand (mostly positive) are not clear at this stage; the difference is rather puzzling.

The quality of the description of the mixing coefficient as a linear function of the charge transfer parameter is illustrated in Figures 7–10 by also showing the linear fits corresponding to the computed Pearson correlation coefficients. As can be seen, in no case are good values for ***x*** obtained for all systems simultaneously for any of the four mixed functionals described by these figures. The best linear fits were obtained for the SRP***x***sol DF, and in this case the linear function yields reasonable predictions of ***x*** for all H_2_-metal systems but the H_2_ + Ag(111) system (Figure 8). In all cases (Figures 7–10) the linear fits of the CH_4_ + metal surface systems perform poorly at predicting the mixing coefficient for CH_4_ + Ni(100). The analysis in terms of the linear fits thus provides further evidence that it may be worthwhile to revisit the H_2_ + Ag(111) and CH_4_ + Ni(100) systems in order to hopefully obtain better reference values of the barrier heights for these systems.

## 4 Conclusions and Outlook

We have investigated the tunability of several expressions for mixed density functionals in which mixing parameter ***x*** can be tuned to enable the mixed DF to reproduce the reference value of the barrier height to dissociative chemisorption of a molecule on a metal surface. The mixed functionals are tested on the barriers collected in the database we call SBH16, which is equal to the previous SBH17 database in ref (8) with the H_2_ + Pt(211) system removed from it.

Increasing the fraction of RPBE exchange incorporated into the mixed DFs leads to higher barriers. All mixed DFs tested are well-tunable toward higher barriers, as their limiting forms (RPBE, RPBE-vdW1, and RPBE-vdW2) all systematically overestimate the barrier height for the systems in the SBH16 database. It turns out that the biggest challenge to finding a perfectly tunable mixed DF for describing the SBH16 database is to obtain a mixed DF expression with a good lower-energy form, which consistently underestimates barrier heights for systems such as those present in SBH16. This goal is fully met with the mixed SRP***x***sol DF that uses PBE correlation and a mixture of PBEsol and RPBE exchange. The mixed SRP***x***sol-vdW2 DF could describe the minimum barrier height of 15 of the 16 systems using the vdW-DF2 correlation, while the mixed SRP***x***-vdW1 DF could do so for 14 of the 16 systems using the vdW-DF1 correlation. Being able to use mixed DFs with different correlation functionals may be important to obtaining an SRP DF for a particular system because reproducing the minimum barrier height is a necessary but not a sufficient condition for reproducing measured sticking (or dissociative chemisorption) probabilities, as now used for validating SRP functionals and barrier heights: It is also necessary to provide a description of how the barrier height varies when the molecule’s impact site on the surface and its orientation relative to the surface is changed, and this variation may depend strongly on the correlation functional used.^7,14,85^

We also tested whether and how the mixing coefficient of the mixed DFs is correlated with the charge transfer parameter describing the system, i.e., the difference between the work function of the metal surface and the electron affinity of the molecule. The answer depends on which mixed DF is used. For the SRP***x*** and SRP***x***sol DFs, which both use PBE correlation, we found that the optimum fraction of RPBE exchange decreases with the charge transfer parameter, as could be expected on the basis of earlier results. However, the opposite relationship and weaker correlation were found for the mixed DFs using vdW-DF1 or vdW-DF2 correlation. The reason for this difference is not clear.

The results presented here point to several new lines of research. First of all, the results underscore the need to obtain better reference values for the H_2_ + Ag(111), CH_4_ + Ru(0001), and CH_4_ + Ni(100) systems. As noted, the H_2_ + Ag(111) system is among the few systems for which the optimum fraction of RPBE exchange could not be obtained through interpolation with the SRP***x***sol-vdW2 and SRP***x***-vdW1 DFs, which otherwise performed quite well at reproducing minimum barrier heights for the systems in the SBH17 database. Furthermore, the Pearson correlation coefficients describing the relationship between the mixing parameters of the SRP***x*** and SRP***x***sol DFs and the charge transfer parameter took on values more in line with their expected behavior if the results for H_2_ + Ag(111), CH_4_ + Ni(100), and CH_4_ + Ru(0001) were discarded.

A small improvement over using the SRP***x***sol mixed DF could be to use a DF that simply mixes the RPBE and PBEsol exchange-correlation functionals. It is also necessary to provide a description of how the barrier height varies when the molecule’s impact site on the surface and its orientation relative to the surface is changed. This usually does not present a problem, as DFT appears to be rather good at describing the variation of barrier height with geometry. This is also attested by the previous success with developing SRP^7^ and by the comparison of DFT with diffusion Monte Carlo results for H_2_ + Al(110).^28^ However, for systems with a deep van der Waals well,^85^ or systems with a shallow well but an early barrier,^14^ this variation is best described by including a correlation functional approximately describing the van der Waals interaction.

When it comes to designing mixed functionals incorporating a vdW-DF1 or vdW-DF2 correlation, another idea worth testing might be to investigate mixtures of weakly repulsive GGA exchange DFs that are appropriate matches for the vdW1 and vdW2 correlation functionals with the rather repulsive^112^ exchange functionals combined with these C functionals in the original vdW-DF1^31^ and vdW-DF2^32^ DFs. Examples of such exchange functionals have been incorporated in the C09^113^ and CX^114^ vdW functionals and other exchange functionals mentioned in ref (112). Another idea would be to explore mixtures of repulsive meta-GGA DFs (such as MS-B86bl^34^) and attractive meta-GGA DFs (such as SCAN^115^) that tend to overestimate, respectively, underestimate barriers to dissociative chemisorption of molecules on metals.^8^ It would also be of interest to investigate the performance of mixtures of, or parametrized forms of, screened hybrid functionals such as HSE06^116^ and screened hybrid functionals incorporating van der Waals correlation.^112,117^ However, it might be most productive to test such hybrid functionals once a database becomes available that also incorporates good reference values of barrier heights for systems characterized by charge transfer parameters <7 eV, such as O_2_ + Ag(111)^35^ and HCl + Au(111).^118^ Such systems presently defy an accurate description based on DFs incorporating GGA exchange.^35,118,119^

## Supporting Information Available

Equilibrium lattice constants calculated with the SRP***x*** and the SRP***x***sol DFs for Ag, Cu, Ir, Pt, and Ru (Table S1). Barrier heights computed with the PBE, the RPBE, the PBEsolc, the PBE-vdW1, the PBE-vdW2, the PBEα-vdW1, the RPBE-vdW1, the RPBE-vdW2, and the PBEsol-vdW2 for 16 molecule-metal surface systems (Tables S2 & S3 and Figures S1–S4). Optimal mixing coefficient ***x*** for SRP***x***, SRP***x***sol, SRP***x***-vdW1, SRP***x***-vdW2, SRP***x***sol-vdW2, SRP***x***-vdW1-ext, and SRP***x***-vdW2-ext (Tables S4 & S5) (PDF)
