# Supplementary material for: Constructing Mixed Density Functionals for Describing Dissociative Chemisorption on Metal Surfaces: Basic Principles
Source: J Phys Chem A. 2023 Dec 5;127(49):10481–98. doi: 10.1021/acs.jpca.3c01932 (PMC10726370; doi:10.1021/acs.jpca.3c01932)
Supplement: Supplementary file 1 — jp3c01932_si_001.pdf [file jp3c01932_si_001.pdf]

Supporting Information for : Constructing Mixed Density Functionals for Describing Dissociative Chemisorption on Metal Surfaces: Basic Principles.

Théophile Tchakoua,† Tim Jansen,† Youri van Nies,† Rebecca F. A. van den Elshout ,‡ Bart A. B. van Boxmeer,¶ Saskia P. Poort,‡ Michelle G. Ackermans,‡ Gabriel Spiller Beltrão,‡ Stefan A. Hildebrand,† Steijn E. J. Beekman,† Thijs van der Drift,† Sam Kaart,† Anthonie Šantić,† Esmee E. Spuijbroek,‡ Nick Gerrits,† Mark F. Somers,† and Geert-Jan Kroes*,†

†*Leiden Institute of Chemistry, Gorlaeus Laboratories, Leiden University, P.O. Box 9502, 2300 RA Leiden, The Netherlands*

‡*Department of Chemical Engineering, Delft University of Technology, 2629 HZ Delft, The Netherlands*

¶*University of Antwerp, Faculty of Applied Engineering, Groenenborgerlaan 171, 2020 Antwerpen, Belgium*

E-mail: *g.j.kroes@chem.leidenuniv.nl

Phone: +31 71 527 4396

Table **S1**: Measured zero-point-energy-corrected and computed equilibrium lattice constants a of the fcc metals Ag, Cu, Ir, Ni, and Pt, and a and c of the hcp metal Ru are presented. The computed values have been calculated with the SRP$\boldsymbol{x}$ and SRP$\boldsymbol{x}$sol DFs varying $\boldsymbol{x}$ by steps $\Delta\boldsymbol{x}$ of 0.1.

Metal	Ag	Cu	Ir	Ni	Pt	Ru	
	a	a	a	a	a	a	c
Experimental	4.062[1]	3.597[1]	3.831[1]	3.499[1]	3.912[1]	2.703[2]	4.274[2]
			SRP$\boldsymbol{x}$				
PBE	4.147	3.635	3.873	3.518	3.968	2.721	4.293
$\boldsymbol{x}$=0.1	4.153	3.639	3.874	3.521	3.970	2.722	4.295
$\boldsymbol{x}$=0.2	4.159	3.644	3.875	3.524	3.972	2.723	4.297
$\boldsymbol{x}$=0.3	4.165	3.648	3.877	3.528	3.974	2.725	4.299
$\boldsymbol{x}$=0.4	4.172	3.652	3.878	3.531	3.976	2.726	4.301
$\boldsymbol{x}$=0.5	4.178	3.657	3.880	3.535	3.979	2.727	4.303
$\boldsymbol{x}$=0.6	4.185	3.661	3.882	3.539	3.981	2.728	4.305
$\boldsymbol{x}$=0.7	4.191	3.666	3.883	3.542	3.983	2.730	4.307
$\boldsymbol{x}$=0.8	4.198	3.670	3.885	3.546	3.985	2.731	4.308
$\boldsymbol{x}$=0.9	4.205	3.675	3.886	3.550	3.988	2.732	4.310
RPBE	4.213	3.679	3.888	3.553	3.990	2.733	4.312
			SRP$\boldsymbol{x}$sol				
PBEsol	4.035	3.559	3.822	3.454	3.902	2.683	4.237
$\boldsymbol{x}$=0.1	4.051	3.570	3.828	3.464	3.910	2.688	4.244
$\boldsymbol{x}$=0.2	4.067	3.581	3.834	3.473	3.918	2.693	4.252
$\boldsymbol{x}$=0.3	4.083	3.593	3.841	3.482	3.927	2.698	4.260
$\boldsymbol{x}$=0.4	4.099	3.604	3.847	3.492	3.935	2.704	4.268
$\boldsymbol{x}$=0.5	4.117	3.616	3.854	3.502	3.944	2.709	4.275
$\boldsymbol{x}$=0.6	4.134	3.628	3.860	3.512	3.953	2.714	4.283
$\boldsymbol{x}$=0.7	4.152	3.641	3.867	3.522	3.962	2.719	4.290
$\boldsymbol{x}$=0.8	4.172	3.653	3.874	3.532	3.971	2.723	4.298
$\boldsymbol{x}$=0.9	4.192	3.666	3.881	3.543	3.980	2.728	4.305
RPBE	4.213	3.679	3.888	3.553	3.990	2.733	4.312

Table **S2**: Calculated barrier heights E_b computed with limiting forms of the mixed DFs are shown for the 16 systems present in the SBH16 database, as calculated for the PBE, the RPBE, the PBEsolc, the PBE-vdW1, the PBE-vdW2, and the PBEα-vdW1 DF with α=0.57. Also presented are the values of the charge excitation parameter ΔE_{CT}=WF-EA. The systems are arranged with the charge transfer parameter increasing from top to bottom.

System	E_b^{PBE}	E_b^{RPBE}	$E_b^{PBEsolc}$	$E_b^{PBE-vdW1}$	$E_b^{PBE-vdW2}$	$E_b^{PBE\alpha-vdW1}$	WF-EA
N_2+Ru(10$\bar{1}$0)	-0.096	0.469	-0.999	-0.023	0.247	-0.314	6.582
N_2+Ru(0001)	1.532	1.965	0.896	1.688	1.746	1.314	7.382
H_2+Ag(111)	1.132	1.457	0.643	1.442	1.569	1.275	7.685
H_2+Cu(110)	0.639	0.874	0.346	0.914	0.996	0.792	7.715
H_2+Cu(100)	0.584	0.905	0.095	0.894	1.024	0.731	7.885
H_2+Cu(111)	0.463	0.760	0.026	0.771	0.886	0.617	8.055
H_2+Ni(111)	0.026	0.170	-0.097	-0.006	0.085	-0.076	8.395
H_2+Ru(0001)	0.014	0.108	-0.050	-0.049	0.002	-0.096	8.555
H_2+Pt(111)	0.018	0.169	-0.103	-0.005	0.063	-0.079	9.065
CH_4+Ni(211)	0.675	0.973	0.349	0.603	0.777	0.448	10.72
CH_4+Ni(100)	0.912	1.259	0.491	0.843	1.020	0.664	10.92
CH_4+Ni(111)	1.010	1.349	0.594	0.962	1.156	0.785	10.99
CH_4+Ru(0001)	0.856	1.164	0.456	0.760	0.989	0.603	11.15
CH_4+Pt(211)	0.489	0.789	0.092	0.484	0.618	0.316	11.39
CH_4+Ir(111)	0.875	1.186	0.495	0.714	0.894	0.550	11.53
CH_4+Pt(111)	0.819	1.151	0.414	0.775	0.898	0.604	11.66

Table **S3**: Calculated barrier heights E_b computed with limiting forms of the mixed DFs are shown for the 16 systems present in the SBH16 database, as calculated for the the PBEα-vdW1 DF with α=0.57, the RPBE-vdW1 DF, the RPBE-vdW2 DF, and the PBEsol-vdW2 DF. Also presented are the values of the charge excitation parameter ΔE_{CT}=WF-EA. The systems are arranged with the charge transfer parameter increasing from top to bottom.

System	$E_b^{PBE\alpha-vdW2}$	$E_b^{RPBE-vdW1}$	$E_b^{RPBE-vdW2}$	$E_b^{PBEsol-vdW2}$	WF-EA
N_2+Ru(10$\bar{1}$0)	-0.044	0.525	0.801	-0.568	6.582
N_2+Ru(0001)	1.529	2.133	2.367	1.113	7.382
H_2+Ag(111)	1.403	1.758	1.885	1.114	7.685
H_2+Cu(110)	0.887	1.144	1.239	0.714	7.715
H_2+Cu(100)	0.859	1.205	1.332	0.564	7.885
H_2+Cu(111)	0.736	1.064	1.183	0.470	8.055
H_2+Ni(111)	-0.002	0.134	0.209	-0.045	8.395
H_2+Ru(0001)	-0.044	0.043	0.095	-0.052	8.555
H_2+Pt(111)	-0.012	0.145	0.211	-0.044	9.065
CH_4+Ni(211)	0.613	0.907	1.076	0.433	10.72
CH_4+Ni(100)	0.829	1.188	1.352	0.607	10.92
CH_4+Ni(111)	0.949	1.305	1.473	0.713	10.99
CH_4+Ru(0001)	0.775	1.063	1.235	0.565	11.15
CH_4+Pt(211)	0.474	0.790	0.939	0.232	11.39
CH_4+Ir(111)	0.730	1.030	1.210	0.501	11.53
CH_4+Pt(111)	0.726	1.107	1.262	0.529	11.66

Table **S4**: The optimal mixing coefficient $\boldsymbol{x}$ is shown for the mixed DFs SRP$\boldsymbol{x}$, SRP$\boldsymbol{x}$sol, SRP$\boldsymbol{x}$-vdW1, and SRP$\boldsymbol{x}$-vdW2. Also presented are the values of the charge excitation parameter ΔE_{CT}=WF-EA. The systems are arranged with the charge transfer parameter increasing from top to bottom.

System	WF-EA	SRP$\boldsymbol{x}$	SRP$\boldsymbol{x}$sol	SRP$\boldsymbol{x}$-vdW1	SRP$\boldsymbol{x}$-vdW2
N_2 + Ru(10$\bar{1}$0)	6.582	0.88	0.95	0.77	0.27
N_2 + Ru(0001)	7.382	0.71	0.88	0.34	0.01
H_2 + Ag(111)	7.685	-0.15	0.53	-1.14	-1.55
H_2 + Cu(110)	7.715	0.64	0.84	-0.55	-0.91
H_2 + Cu(100)	7.885	0.49	0.79	-0.49	-0.91
H_2 + Cu(111)	8.055	0.55	0.82	-0.49	-0.88
H_2 + Ni(111)	8.395	-0.02	0.45	0.21	-0.42
H_2 + Ru(0001)	8.555	-0.09	0.34	0.57	0.02
H_2 + Pt(111)	9.065	-0.17	0.34	-0.02	-0.48
CH_4 + Ni(211)	10.72	0.08	0.57	0.32	-0.24
CH_4 + Ni(100)	10.92	-0.44	0.34	-0.24	-0.76
CH_4 + Ni(111)	10.99	0.01	0.56	0.27	-0.26
CH_4 + Ru(0001)	11.15	-0.18	0.48	0.13	-0.44
CH_4 + Pt(211)	11.39	0.23	0.67	0.32	-0.14
CH_4 + Ir(111)	11.53	-0.12	0.49	0.39	-0.18
CH_4 + Pt(111)	11.66	0.00	0.54	0.24	-0.17

Table **S5**: The optimal mixing coefficient $\boldsymbol{x}$ is shown for the mixed DFs SRP$\boldsymbol{x}$sol-vdW2, SRP$\boldsymbol{x}$-vdW1-ext, and SRP$\boldsymbol{x}$-vdW2-ext. Also presented are the values of the charge excitation parameter ΔE_{CT}=WF-EA. The systems are arranged with the charge transfer parameter (see Table **S4**) increasing from top to bottom.

System	SRP$\boldsymbol{x}$sol-vdW2	SRP$\boldsymbol{x}$-vdW1-ext	SRP$\boldsymbol{x}$-vdW2-ext
N_2 + Ru(10$\bar{1}$0)	0.70	0.77	0.27
N_2 + Ru(0001)	0.66	0.34	0.01
H_2 + Ag(111)	-0.07	-0.90	-1.21
H_2 + Cu(110)	0.12	-0.46	-0.76
H_2 + Cu(100)	0.78	-0.42	-0.74
H_2 + Cu(111)	0.21	-0.42	-0.72
H_2 + Ni(111)	0.25	0.21	-0.31
H_2 + Ru(0001)	0.34	0.57	0.02
H_2 + Pt(111)	0.15	-0.06	-0.42
CH_4 + Ni(211)	0.42	0.32	-0.22
CH_4 + Ni(100)	0.19	-0.23	-0.60
CH_4 + Ni(111)	0.45	0.27	-0.21
CH_4 + Ru(0001)	0.67	0.13	-0.38
CH_4 + Pt(211)	0.50	0.32	-0.15
CH_4 + Ir(111)	0.53	0.39	-0.19
CH_4 + Pt(111)	0.55	0.24	-0.21

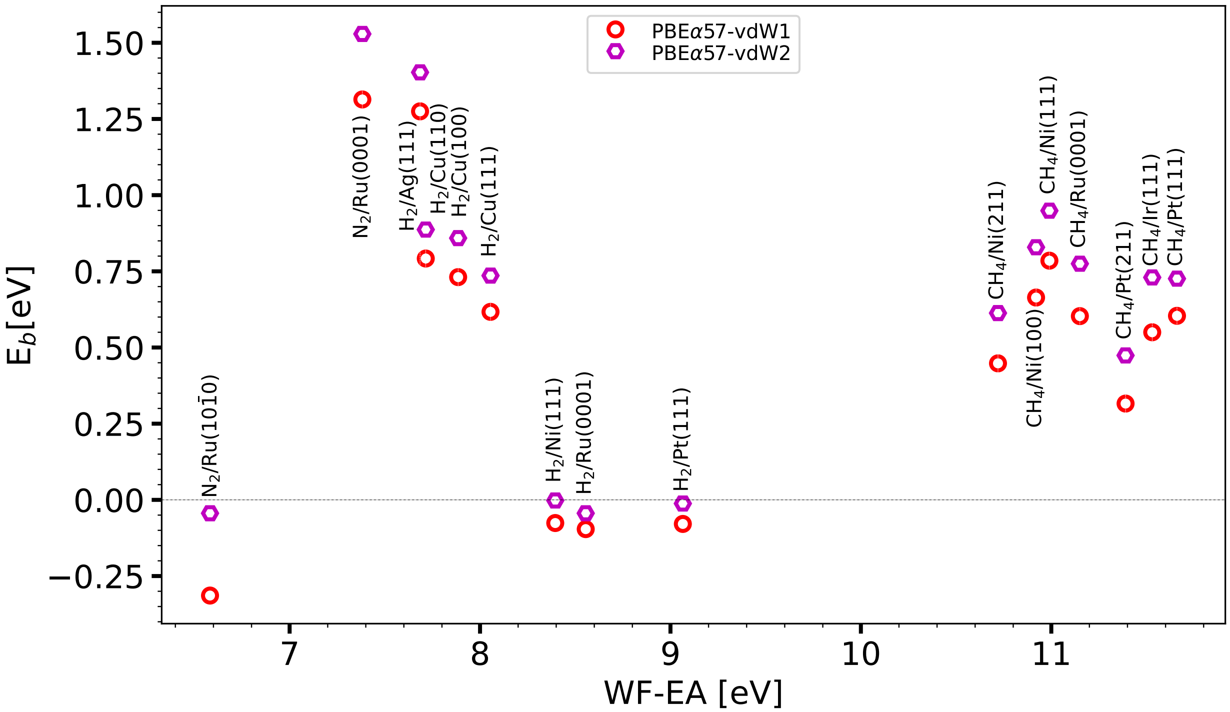

Figure **S1**: The barrier heights E_b computed with the PBEα-vdW1 and PBEα-vdW2 DFs with α=0.57 are shown as a function of the charge transfer parameter for the 16 systems present in the SBH16 database. These DFs may be viewed as the lower-limit expressions given by Eqs. 6b and 7b, respectively.

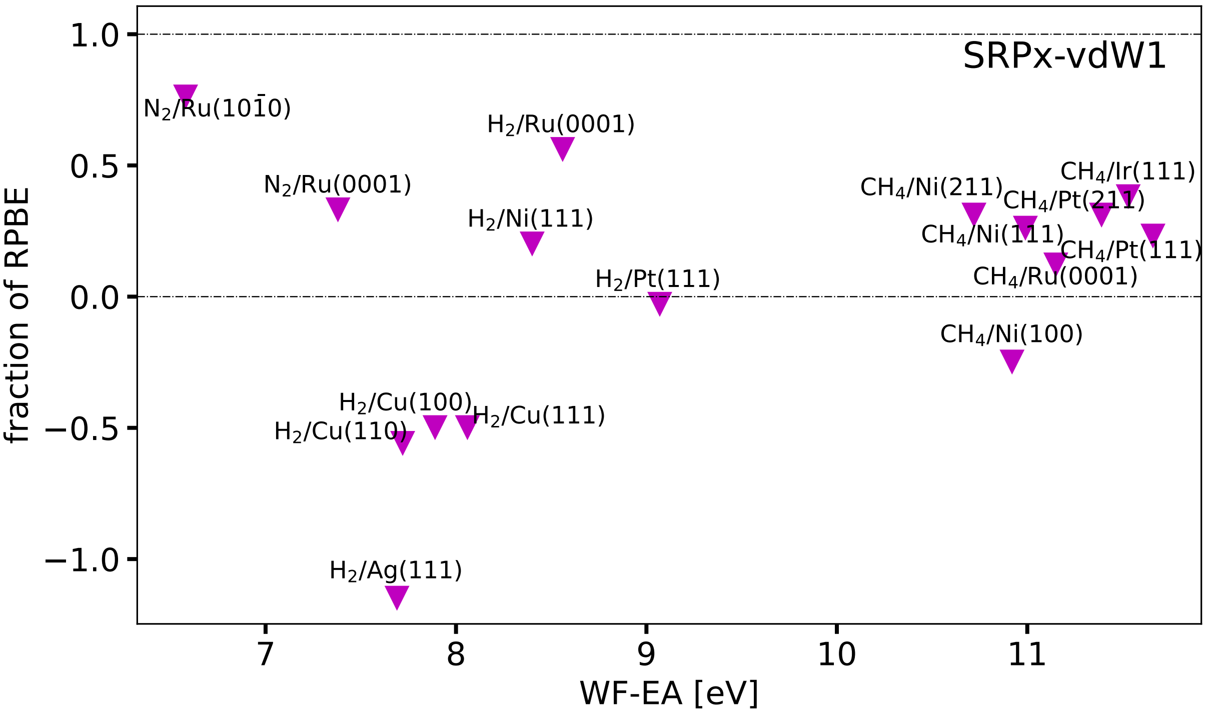

Figure **S2**: The optimum fraction of RPBE exchange $\boldsymbol{x}$ is shown as a function of ΔE_{CT} for the SRP$\boldsymbol{x}$-vdW1 DF (Eq.3). Values falling between the two horizontal dot-dashed black lines could be obtained by the interpolation procedure illustrated in Figure 5.

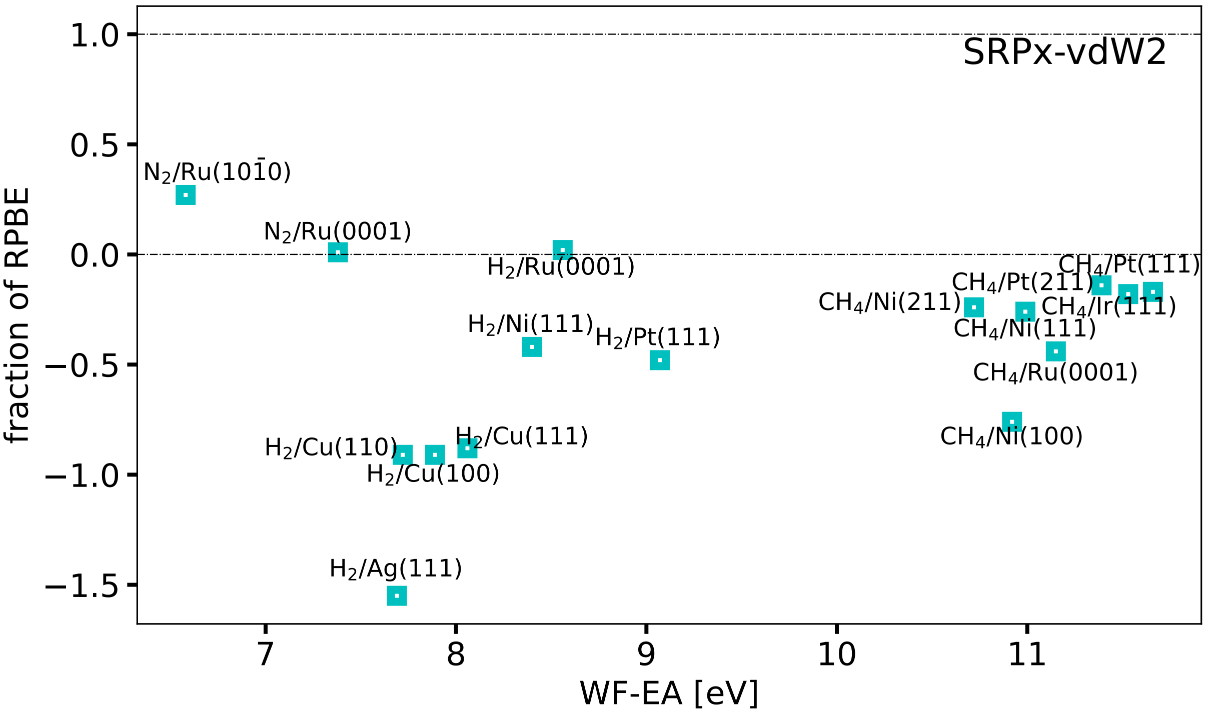

Figure **S3**: The optimum fraction of RPBE exchange $\boldsymbol{x}$ is shown as a function of ΔE_{CT} for the SRP$\boldsymbol{x}$-vdW2 DF (Eq.4). Values falling between the two horizontal dot-dashed black lines could be obtained by the interpolation procedure illustrated in Figure 5.

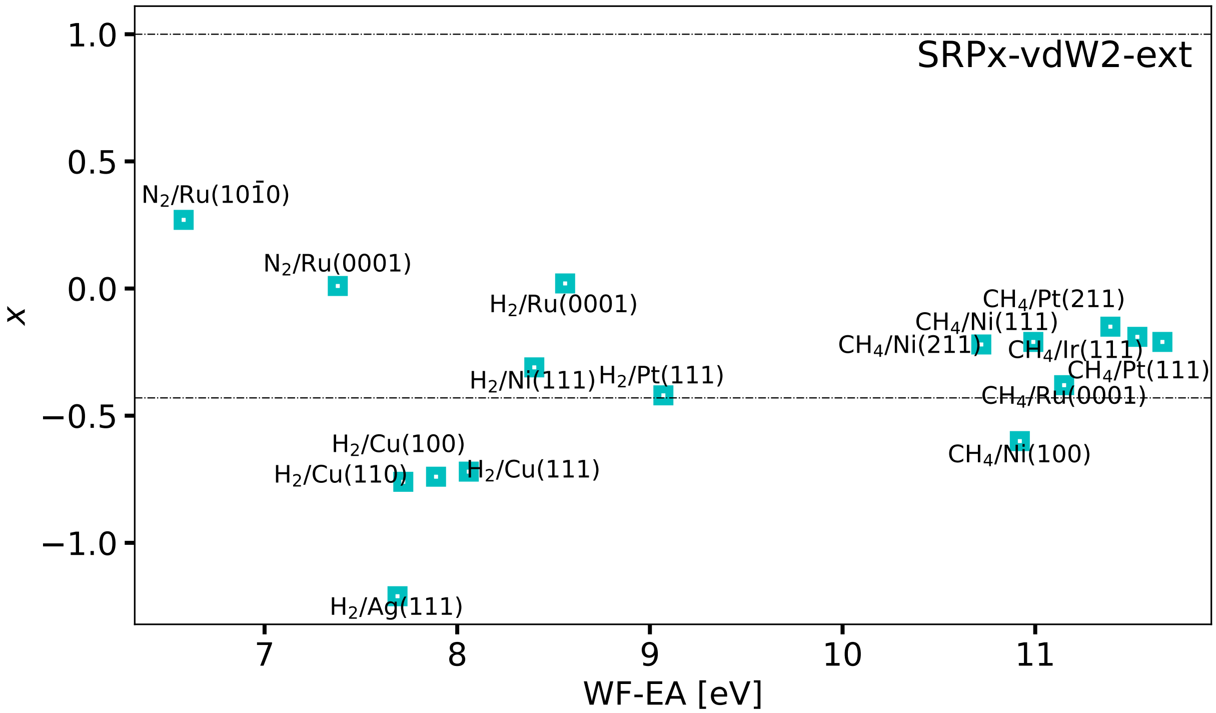

Figure **S4**: The optimum mixing parameter $\boldsymbol{x}$ is shown as a function of ΔE_{CT} for the SRP$\boldsymbol{x}$-vdW2-ext DF (Eq.7). Values falling between the two horizontal dot-dashed black lines could be obtained by the interpolation procedure illustrated in Figure 5.

References

(1) Haas, P.; Tran, F.; Blaha, P. Calculation of the lattice constant of solids with semilocal functionals. *Phys. Rev. B* **2009**, *79*, 085104.

(2) Arblaster, J. W. Crystallographic properties of ruthenium. *Platinum Met. Rev.* **2013**, *57*, 127–136.
